# Isopotential Electron Titration: Hydrogen Adsorbate-Metal Charge Transfer

**DOI:** 10.1021/acscentsci.5c00851

**Published:** 2025-09-15

**Authors:** Justin A. Hopkins, Benjamin J. Page, Shengguang Wang, Jesse R. Canavan, Jason A. Chalmers, Susannah L. Scott, Lars C. Grabow, James R. McKone, Paul J. Dauenhauer, Omar A. Abdelrahman

**Affiliations:** † Center for Programmable Energy Catalysis, University of Minnesota, Department of Chemical Engineering & Materials Science, 421 Washington Ave. SE, Minneapolis, Minnesota 55455, United States; ‡ Department of Chemical Engineering & Materials Science, 5635 University of Minnesota, 421 Washington Ave. SE, Minneapolis, Minnesota 55455, United States; § William A. Brookshire Department of Chemical and Biomolecular Engineering, 14743 University of Houston, 4226 Martin Luther King Blvd., Houston, Texas 77204, United States; ∥ Texas Center for Superconductivity, 14743 University of Houston, 4226 Martin Luther King Blvd., Houston, Texas 77204, United States; ⊥ Department of Chemical Engineering, 8786 University of California, Santa Barbara, California 93106, United States; # Department of Chemical and Petroleum Engineering, 6614 University of Pittsburgh, 3700 O’Hara St, Pittsburgh, Pennsylvania 15213, United States

## Abstract

The extent of charge transfer between an adsorbate and thermocatalytic surface plays a key role in determining catalytic activity, but direct and quantitative measures have remained elusive. Here, we report the method of isopotential electron titration (IET), an approach that directly measures charge transfer between adsorbates and catalytic surfaces. Charge transfer between Pt and adsorbed hydrogen adatoms was investigated using a catalytic condenser, where the Pt surface was separated from a p-type silicon layer by a hafnia dielectric film. By forcing the Pt and Si layers into isopotential conditions, charge transfer between the adsorbate and Pt surface was titrated through an external circuit. Hydrogen atoms donated electrons to Pt upon adsorption, which was quantitatively reversed upon desorption. Across a temperature range of 125–200 °C (surface hydrogen fractional coverages of 80–100%), the charge transferred to Pt by an adsorbed hydrogen atom was measured to be 0.19 ± 0.01% |e|/H. Bader charge analysis of the extent of charge transfer was in agreement with experimental measurements, with a calculated net donation of 0.4% |e|/H. The ability to experimentally quantify surface charge transfer provides an electronic-based approach to characterize catalytic surfaces, the adsorbed moieties residing on them, and the chemical reactions they accelerate.

## Graphical abstract

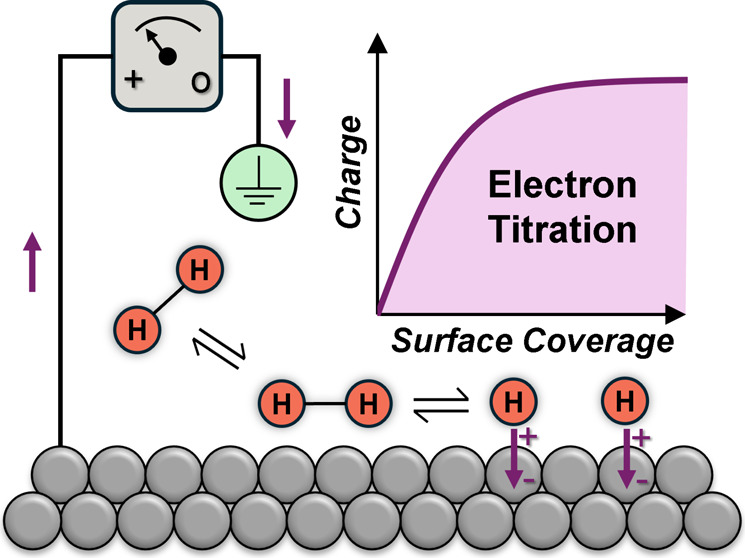

## 1 Introduction

Heterogeneous catalysis relies on the ability of a solid surface to lower the activation energy of a chemical reaction by forming bonds with adsorbates, through the exothermic transfer of electrons that energetically favors the partitioning of adsorbates on a surface, relative to a bulk fluid.^1^ The ability of a catalyst to transfer electrons to or from a bonded adsorbate (via Lewis acid–base interactions,^2–6^ or the *d*-band center for transition metal catalysts^7–9^ ) is reflected in the energy of a surface–adsorbate bond relative to the initial unbound states (i.e., the binding energy), which has served as an important descriptor of catalytic activity.^10–13^

Charge transfer is not unique to heterogeneous catalysis. In homogeneous catalysis, the oxidation state of a metal center changes in the course of the cycle, as the coordination number of the metal center changes (e.g., hydrogenation by Wilkinson’s catalyst^14,15^ ). Electrocatalysis externally manipulates the electrochemical potential gradient across an electrode/electrolyte interface to drive electron transfer processes,^16,17^ altering the reaction Gibbs free energy to facilitate otherwise nonspontaneous reactions at appreciable net rates.^16–18^ In general, the modulation of the Gibbs free energy of a reaction by charge-potential work can be described by eq 1,

$$\frac{\partial G^{o}}{\partial \chi }={\mathrm{dG}}_{\Delta \varphi =0}^{o}+\mathrm{\delta F}\Delta \varphi$$ 1

where $\frac{\partial G^{o}}{\partial \chi }$ is the differential Gibbs free energy at standard state with respect to the extent of reaction (in moles, where the extent of reaction can be an adsorption event, a surface reaction, or an entire catalytic cycle), dG_Δφ=0_^o^ is the molar Gibbs free energy change for the reaction at standard state at zero applied electric field (Δφ = 0), δ is the extent of charge transfer along the reaction coordinate (δ > 0, surface species transfer electrons to the surface), F is Faraday’s constant, and Δφ is the potential difference between adsorbate and catalyst surface (Δφ > 0, higher adsorbate potential). If the extent of charge transfer from an adsorbing molecule is nonzero (i.e., a dipole moment between adsorbate and surface forms, Figure 1A), then an applied potential across the adsorbate-metal interface introduces electrostatic work into the system that alters adsorbate thermodynamic stability.^19,20^ The work supplied to or extracted from the system along the reaction coordinate (W) is the product of the charge transferred, Faraday’s constant (F), and the adsorbate–surface potential difference, as shown in eq 2:

$$W=\mathrm{\delta F}\Delta \varphi \,\;[=]\;J\,\;{\mathrm{mol}}^{-1}$$ 2

**Figure 1:**
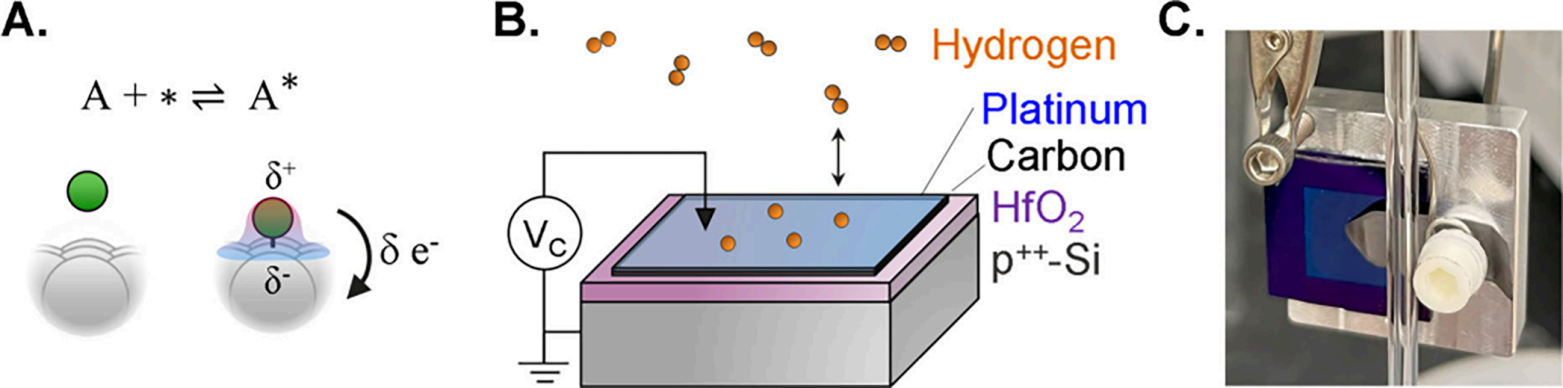
**A**. Adsorption event involving charge transfer between an adsorbate and catalyst site. **B**. Hydrogen (orange) adsorption on a catalytic condenser schematic, p^++^-Si (gray), HfO_2_ insulator (purple), carbon conducting layer (black), and Pt (blue) with an applied potential (V_c_). **C**. Picture of Pt/C/HfO_2_/p^++^-Si device, 1 cm^2^ in size.

This electrostatic work can cause the overall Gibbs’ free energy change to become negative for the reaction event (i.e., $\frac{\partial G^{o}}{\partial \chi }<0$ ), facilitating a reaction that would otherwise be nonspontaneous.

Many phenomena induce and perturb electric potential gradients across catalyst surfaces, including supporting a catalyst on a material with a different Fermi energy level^21–26^ and the presence of promoters on the catalyst surface.^27–29^ These potential gradients, intentionally induced or otherwise, influence both the Gibbs free energy change of reaction (e.g., electrocatalytic processes) and the Gibbs free energy of a surface intermediate or transition state experiencing a nonzero extent of charge transfer (eq 1, δ ≠ 0). Electric fields have been reported to influence the rates of chemical reaction and/or selectivity to products, whether applied directly at a catalyst bed,^30,31^ via an electrode,^32,33^ or by surface plasmons.^34,35^ However, experimental methods have not explicitly measured the extent of charge transfer, focusing instead on the overall catalytic effect of an applied voltage or electric field.

Charge transfer between catalyst surfaces and adsorbates can be probed electrochemically in the presence of a liquid phase,^19,36–38^ but such measurements are complicated by the simultaneous occurrence of large Faradaic currents. Charge transfer between adsorbates and catalytic surfaces in the absence of a liquid phase or an electrode–electrolyte interface are limited, with most studies performed under ultrahigh vacuum (UHV) conditions using ultraviolet photoelectron spectroscopy^39–43^ and Kelvin probe microscopy.^44–46^ These techniques revealed adsorbate induced changes in the work function of the underlying metal surface, suggesting that adsorbates alter the occupancy of frontier electron orbitals near the Fermi energy level at the metal surface (i.e., charge transfer). For example, Christmann, Ertl, and Pignet showed that hydrogen adsorption on Pt(111) lowered the work function of Pt by up to 230 meV,^39^ while Kiskinova, Pirug, and Bonzel reported that adsorbed water lowered the work function of clean Pt(111), but increased it on the K-modified surface.^40^ Despite the importance of charge transfer for catalytic cycles, direct measurements of adsorbate–surface charge transfer under catalytically relevant conditions remains limited.

To measure charge transfer between adsorbates and surfaces, an electronic device capable of measuring and responding to charge storage, such as a capacitor, is needed. We previously described a catalytic condenser in which a nanometer scale catalyst layer is placed on top of a thin film parallel plate capacitor composed of an insulating dielectric and p^++^-Si wafer (Figure 1 **C–D**).^47–51^ Applying a potential across the dielectric layer of the condenser altered the electron density of the catalyst layer, modulating the activation energy for 2-propanol dehydration on Al_2_O_3_ ^47^ and the binding energy of carbon monoxide on Pt.^48–50^ Adsorbate binding energies were found to vary linearly with applied potential, consistent with expected linear change in Gibbs’ free energy for adsorption processes involving a constant extent of charge transfer (eq 1). Combining the reversibility of charge transfer and the ability of a catalytic condenser to influence surface energetics through applied potential, we postulate that catalytic condensers are able to directly measure charge transfer between adsorbates and a catalyst surface.

Here, we demonstrate the functionality of catalytic condensers as an electronic platform for the explicit measurement of charge transfer between adsorbates and surfaces, using a technique called ‘isopotential electron titration’ (IET). By fixing the potential across a catalytic condenser (isopotential), changes in catalyst work function were coupled with electrical current resulting from the titration of electrons transferred during adsorption (i.e., removing an equal number of electrons to those transferred via adsorption). IET was used to investigate charge transfer associated with dissociative hydrogen adsorption on Pt, across a range of catalytically relevant temperatures and pressures. Hydrogen was chosen as the first molecule to study due to its ubiquitous presence and redox nature in catalytic cycles both directly through liquid media^36,38^ or through direct activation on metals such as Pt or Pd.^52,53^ Under all conditions examined in this work, electron removal from Pt upon hydrogen adsorption was necessary to maintain isopotential conditions; hydrogen donated charge to the Pt surface upon adsorption, consistent with a decrease in the Pt work function. By correlating the charge transferred from hydrogen atoms adsorbed with the molar quantity of hydrogen atoms adsorbed, a direct measure of the extent of charge transfer was possible, which was found to be in agreement with the theoretical predictions of a Bader charge analysis of hydrogen atoms adsorbed on Pt. The ability to quantify the magnitude and direction of partial charge transfer involved in the elementary chemical steps under realistic reaction conditions will advance the development of field-dependent approaches to accelerate chemical catalysis by identifying and characterizing reactions responsive to charge-voltage work.

## 2 Results and Discussion

Three replicate catalytic condensers (Pt/C/HfO_2_/p^++^-Si) fabricated by previously reported methods,^48,49^ were used to measure the extent of charge transfer upon hydrogen adsorption in two laboratories (Universities of Houston & Minnesota) to confirm replicability. The top Pt layer of the catalytic condenser served as the working electrode, while the bottom p^++^-Si layer acted as the counter electrode, held at electrochemical equilibrium by applying a zero potential between the two (V_c_ = 0, Figure 1C), which in turn was in direct contact with a surrounding gas phase environment. Molecular hydrogen dissociated to adsorbed hydrogen, as shown in eq 3, with the population of hydrogen on the Pt surface (i.e., fractional coverage, θ_H_) calculated through eq 4 using the partial pressure of hydrogen and the adsorption equilibrium constant (K_ads,H_, described by eq 5). These fractional coverages of hydrogen on the Pt were manipulated through step changes in the gas phase partial pressure of molecular hydrogen (P_H_2__) in equilibrium with Pt.

3 $$H_{2}+2\mathrm{Pt}⇌2\mathrm{PtH}$$

4 $$\theta_{H}=\frac{\sqrt{K_{\mathrm{ads},H_{2}}P_{H_{2}}}}{1+\sqrt{K_{\mathrm{ads},H_{2}}P_{H_{2}}}}$$

5 $$K_{\mathrm{ads},H_{2}}=e^{-{\Delta G}_{\mathrm{ads},H_{2}}^{0}/\mathrm{RT}}$$

where ΔG_ads,H_2__^o^ represents the free energy change for hydrogen adsorption on Pt. Step shifts in gas phase hydrogen partial pressure were applied by cycling between hydrogen rich (1 atm H_2_) and lean (5 × 10^–3^ atm H_2_) conditions at a fixed temperature, while forcing isopotential conditions across Pt and p^++^-Si (Figure 2A). By maintaining isopotential conditions during adsorption–desorption events, the condenser architecture served as a molecular ammeter for charge transferred through the top metal layer, measuring the extent of charge transfer between adsorbed hydrogen atoms and the Pt surface.

**Figure 2:**
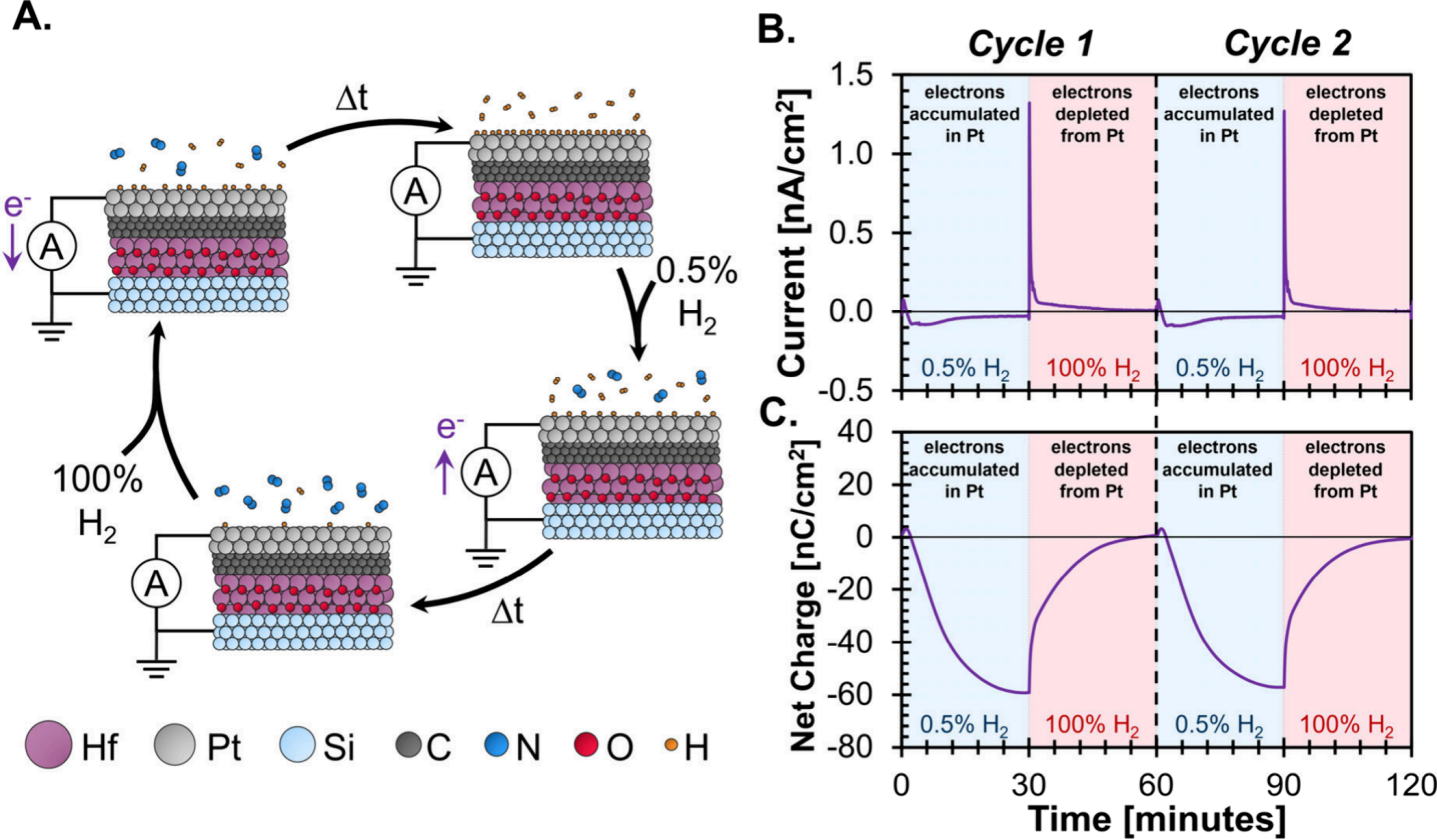
Isopotential electron titration of hydrogen adsorption on Pt. **A**. Pt condenser surface cycling between high and low H* coverage under flow of 100% and 0.5% H_2_, respectively, under isopotential conditions. **B**. Current versus time at 200 °C under isopotential conditions, blue and red shading represents hydrogen-lean and -rich gas phase environments, respectively. **C**. Net charge (eq 7) versus time under isopotential conditions.

Upon switching from a hydrogen-rich to a hydrogen-lean environment at 200 °C, where the fractional coverage of hydrogen on the Pt surface decreased from 98% to 77% (Supporting Information, **Sec. S6 B**), a negative transient current (i.e., electrons accumulated in the Pt) resulting from hydrogen desorption was observed (Figure 2B). A subsequent return to a hydrogen-rich gas phase environment led to a transient current in response to hydrogen adsorbing to repopulate the Pt surface, albeit with opposite polarity (i.e., positive current meaning electrons depleted from the Pt). Control measurements of switches between N_2_ and He (similar thermal conductivity to hydrogen) revealed currents (<1 nA) negligible in magnitude compared to those measured for H_2_ adsorption (1 nA) and in opposing direction, highlighting the minimal contribution of thermoelectric effects. Electrostatic or hydrodynamic effects may have been present at 200 °C, as indicated by control measurements of switches between N_2_ and N_2_, which revealed currents (<1 nA). However, the total contribution of the effect was at most 10% of the total quantified charge, with no significant contribution discernible from noise below 200 °C (Supporting Information, **Sec. S5 B-D**). The positive transient current response to hydrogen adsorption was sharper than the transient negative current response to hydrogen desorption (full-width half max = 0.2 min for adsorption vs 13.1 min for desorption), suggesting that the rate of charge transfer during adsorption is significantly faster. The current in the hydrogen lean conditions reached a steady state baseline that is offset from 0 nA/cm^2^. We believe this is because the shift in electrochemical equilibrium upon adsorption results in slow ion distribution in the HfO_2_ dielectric that is not due to charge transfer from H_2_ but is an indirect electronic response. The observed difference in the rate of charge transfer is consistent with the more favorable kinetics of dissociative hydrogen adsorption relative to associative desorption of H_*_ from a Pt surface, as shown in eq 6.^54^

6 $$K_{\mathrm{ads},H_{2}}=\frac{k_{\mathrm{ads}}}{k_{\mathrm{des}}}=2330{\mathrm{atm}}^{-1}@200^{o}C$$

Repeated transitions between hydrogen rich/lean environments resulted in identical transient current responses, consistent with the reversibility of hydrogen adsorption on a Pt surface (Figure 2B, Cycle 1 and Cycle 2). Similarly, the net charge across a single adsorption–desorption cycle (calculated by integrating the current according to eq 7), representative of the total adsorbate-metal charge transfer, must equal zero for a reversible process (Figure 2C),

7 $$Q_{\mathrm{net}}=Q_{\mathrm{ads}}+Q_{\mathrm{des}}=\int I\left(t\right)\mathrm{dt}$$

The zero net charge across an adsorption–desorption cycle suggests that Pt returned to the same electronic state after each cycle, which is necessary if the response is due to transitions between equilibrated states. The polarity of the transient current responses suggests that hydrogen donated electrons to the Pt surface upon adsorption, accumulating electrons in Pt that were removed through the external circuit of the potentiostat to maintain isopotential conditions. Conversely, electrons were depleted from the Pt layer and transferred back to hydrogen upon desorption. The adsorbate-metal electronic interactions can be captured through a modification of the elementary step convention used to describe adsorption on metal surfaces (eq 3), explicitly accounting for charge-transfer phenomena as shown in eqs 8–10,

8 $$H_{2}⇌2H^{\delta^{+}}+2\delta e^{-}$$

9 $$\mathrm{Pt}+\delta e^{-}⇌{\mathrm{Pt}}^{\delta^{-}}$$

10 $$H_{2}+2\mathrm{Pt}⇌{2\mathrm{Pt}}^{\delta^{-}}H^{\delta^{+}}$$

where adsorbed hydrogen adopts a partial positive charge (H^δ+^) and Pt a partial negative charge (Pt^δ‑^). Previous studies of catalytic nanodiodes reported current flow over a Schottky barrier, which was ascribed to energy conversion from exothermic adsorption to electron–hole pair formation in the metal.^55–57^ However, our results are described by adsorbate-metal charge transfer since current was observed for both adsorption (exothermic) and desorption (endothermic) cycles.

To understand the mechanism through which charge transferred from hydrogen to Pt was detected, we considered the response of the electronic band structure to molecular adsorption under isopotential electron titration. Prior to hydrogen adsorption, the Pt and p^++^-Si surfaces were in electrochemical equilibrium (E_F,Pt_ – E_F, p^++^‑Si_ = 0), with no net electron flow between the two (I = 0, Figure 3A). Once hydrogen adsorbed and donated electrons to the Pt surface (eq 8–10 **)**, the chemical potential of electrons in Pt was increased, decreasing its work function and driving the catalytic condenser out of electrochemical equilibrium (E_F, Pt_ - E_F_,_p^++^‑Si_ > 0, Figure 3B). The decrease in work function is consistent with the results by Christmann, Ertl, and Pignet for conditions of moderate-to-high hydrogen surface coverage on Pt(111), and on Pt 6(111)×(100) by Collins and Spicer.^39,41^ However, given that Pt and p^++^-Si were also connected via the external circuit of a potentiostat forcing a zero potential difference between them, the accumulated electrons were removed to restore electrochemical equilibrium, resulting in a measurable transient current (I > 0). Once electrochemical equilibrium between p^++^-Si and hydrogen-covered Pt was achieved, no further net flow of electrons was possible (I = 0, Figure 3 **C)**. The same logic rationalizes the negative current observed upon hydrogen desorption; Pt donates charge back to desorbing hydrogen and electrons flow from the p^++^-Si layer through the external circuit to reestablish electrochemical equilibrium, equal in amount to those transferred from Pt during hydrogen adsorption (Figure 2C, eq 7).

**Figure 3:**
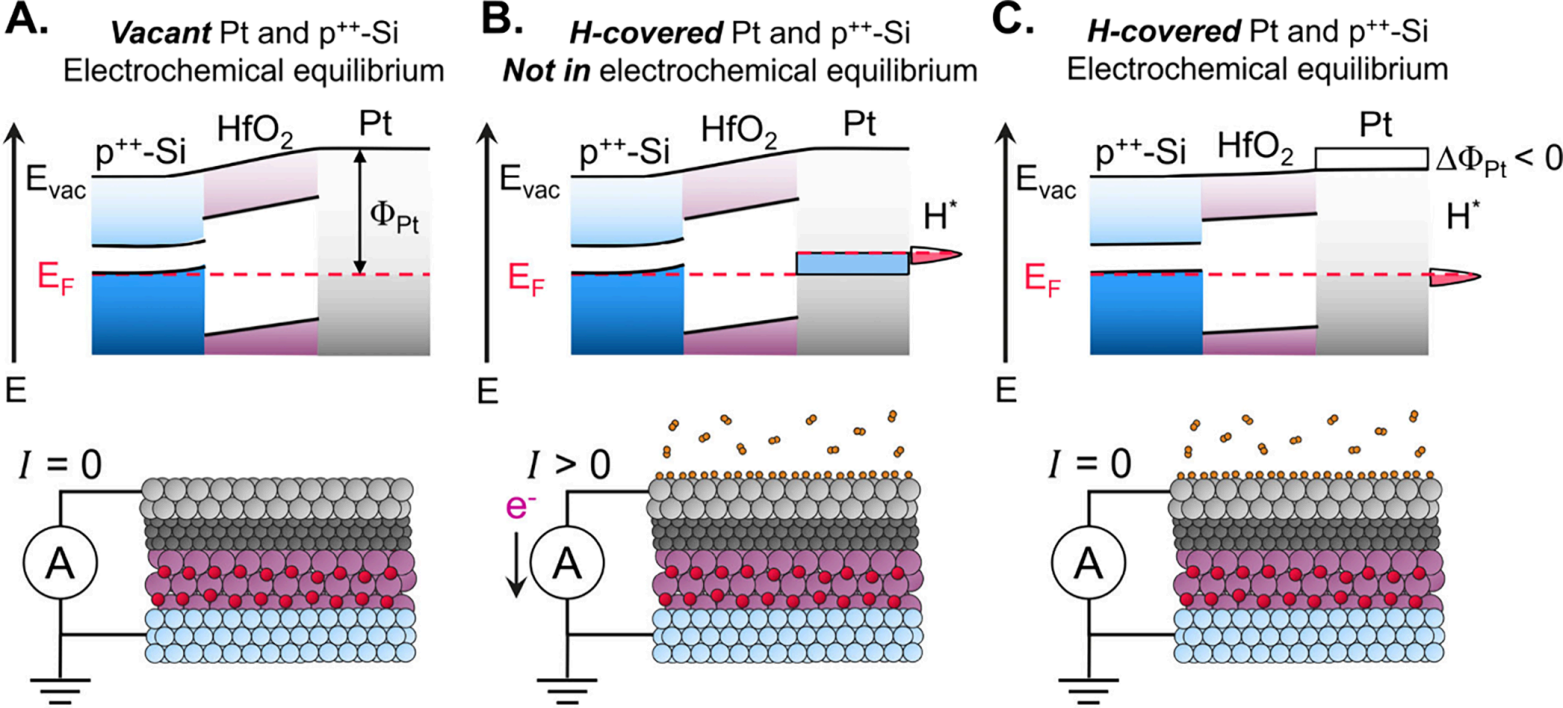
Catalytic condenser electronic band structure during isopotential electron titration. **A**. Vacant Pt surface in electrochemical equilibrium with p^++^-Si with no net electron flow (I = 0). Pt work function (Φ_Pt_) is the difference between the Fermi level (E_f_, red dashed line) and the vacuum level (E_vac_, solid black line). **B**. Surface states filled by adsorbed hydrogen (H_*_) lower the work function of Pt, net electron flow from Pt to p^++^-Si to re-establish equilibrium (I > 0). **C**. Equilibrium between hydrogen-covered Pt and p^++^-Si, no net electron flow (I = 0).

Having manipulated the surface population of adsorbing hydrogen through changes in hydrogen partial pressure, we considered the influence of temperature on the extent of charge transfer. Given the exothermic nature of hydrogen adsorption on Pt (ΔH_ads_^0^ = −64 kJ mol^–1^),^58^ the fractional coverage of surface hydrogen (θ_H_) in equilibrium with a fixed partial pressure of hydrogen in the bulk, decreases with increasing temperature (Figure 4A). As a result, greater changes in hydrogen fractional coverage (Δθ_H_) in response to cycling between hydrogen rich (1 atm H_2_) and lean (5 × 10^–3^ atm H_2_) environments were experienced with increasing temperature (Δθ_H_, Figure 4B). Details of estimating the hydrogen surface coverage on Pt are provided in the Supporting Information (**Sec. S6**). As expected from greater shifts in the amount of adsorbed hydrogen, the total quantity of charge transferred to and from Pt during hydrogen adsorption and desorption, respectively, increased with temperature (125 to 200 °C, Figure 4C). However, independent of temperature, equal amounts of charge were transferred to and from the Pt surface during hydrogen adsorption and desorption, respectively (eq 7). Additionally, a consistent average charge of adsorption/desorption was measured across multiple condenser devices, laboratories, and reactors, demonstrating the reproducibility of IET measurements (Figure 4D).

**Figure 4:**
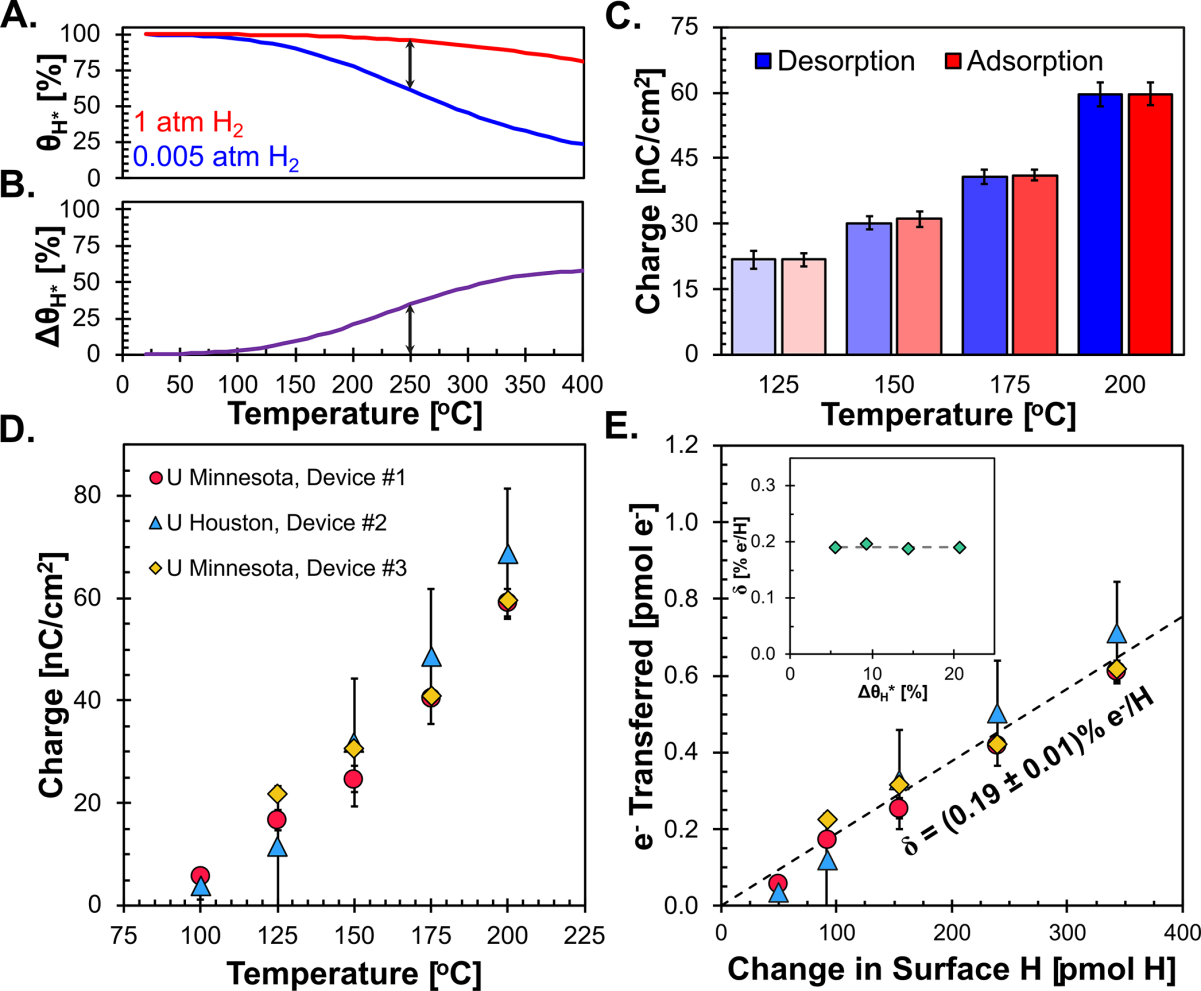
Charge transfer between hydrogen and Pt across temperature. **A**. Calculated fractional coverage of hydrogen (H_*_) on Pt as a function of temperature for hydrogen rich (1 atm, red) and lean (5 × 10^–3^ atm, blue) environments. **B**. Change in H* fractional coverage between hydrogen rich and lean isobars. **C**. Absolute measured charge for hydrogen adsorption (red) or desorption (blue) at isopotential conditions for 125, 150, 175, and 200 °C. **D**. Average of measured adsorption/desorption charge as a function of temperature for three devices: Device #1 (red circle), Device #2 (blue triangles), Device #3 (yellow diamonds). **E**. Measured electrons transferred as a function H atoms adsorbed/desorbed from Pt and extent of charge transfer as a function of coverage change (inset).

To ascribe the measured apparent charge transfer to the extent of charge transfer (δ, eq 1), the electrons titrated under isopotential conditions must be equal to the actual amount of charge transferred from H* to Pt upon bond formation. Given the minimal loading of Pt relative to that of p^++^-Si, the total number of charge carriers in Pt was far outnumbered by that of p^++^-Si (Supporting Information, **Sec. S9**).^59,60^ As a result, the electronic states of the grounded p^++^-Si are relatively unperturbed by the charge transferred to or from it, serving as an electronic sink or source when hydrogen adsorbs or desorbs from Pt, respectively. This type of behavior was observed in other catalytic systems where the metal catalyst is scarce compared to a semiconductor.^22,26^ Therefore, we considered that all electrons titrated under the forced isopotential condition (N_e_, calculated by eq 11) originated from hydrogen transferring charge to Pt upon adsorption,

11 $$N_{e}=\frac{Q_{\mathrm{ads}/\mathrm{des}}}{F}$$

which exhibited a linear correlation with the moles of surface hydrogen adsorbed across the various temperatures (Figure 4E). The linearity of the relationship suggested that the charge transferred from adsorbate to surface is relatively temperature insensitive over the range of temperature (125 – 200 °C) and hydrogen coverages considered in this work, with a value of 0.19 ± 0.01% |e|/H of an elementary electronic charge donated per adsorbed surface hydrogen atom, highlighting that the extent of charge transfer remained consistent across the various coverages (inset of Figure 4E). While lateral interactions between adsorbates could result in a coverage dependent extent of charge transfer, across the limited range considered in this work, experimental work function shifts^39^ remained relatively linear with coverage (see Supporting Information, Sec. S6 C for observed work function trends and comparison to theory).

To provide an estimate against which to compare the experimental result, we calculated the expected extent of charge transfer of hydrogen on Pt using a density functional theory (DFT)-based Bader charge analysis. Hydrogen atoms adsorb on both 3-fold hollow sites and atop sites with minimal energetic preference (E_H,Hollow_ ∼ E_H,Atop_) on Pt(111). To quantify charge transfer at the higher coverages representative of the experimental IET measurements (θ_H_ = 0.77 – 0.98), the average net charge per adsorbed hydrogen atom was calculated using Bader charge analysis^61^ for a surface coverage of 29.3 H/nm^2^ (Figure 5 **A)**. While the extent of charge transfer between H* and Pt on 3-fold hollow (5.2% |e|/H) and atop sites (4.4% |e|/H) is similar in magnitude, the direction of charge transfer is different (Figure 5B). Hydrogen atoms donated and accepted charge when adsorbed on 3-fold hollow and atop sites, respectively. Experimentally, it is difficult to determine the site distribution of H* at atmospheric pressures and elevated temperatures. However, ultrahigh vacuum literature has reported that hydrogen prefers to adsorb on 3-fold hollow sites on Pt(111) (e.g., using inelastic neutron^62^). This finding is corroborated by theoretical studies of hydrogen adsorption on platinum as well.^63^ The partial charge of adsorbed hydrogen, therefore, was dependent on Pt adsorption site. To compare theoretically predicted charge transfer to experimental measurements where charge from individually adsorption sites was not distinguishable, the ensemble weighted average of the theoretical charge transfer was considered. The calculated weighted average charge was calculated to be +0.40% |e|/H, comparable to the experimentally measured value of + (0.19 ± 0.01)% |e|/H. It should be noted that while reasonable to assume a Pt(111) facet, the presence of surface defects, and difference in ratios of atop vs hollow sites can readily explain the factor of 2 difference between the computational and experimental extent of charge transfer values.^64^ However, without independent information that motivates considering additional surface/adsorbate structures, Pt(111) provides a reasonable model.

**Figure 5:**
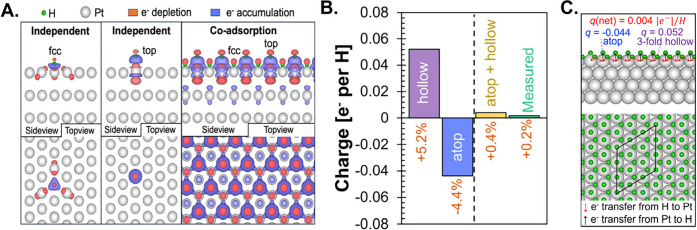
Calculated charge transfer. **A**. Bader charge analysis of hydrogen adsorbed on Pt (111). **B**. Charge on H adsorbed to Pt to 3-fold hollow sites (purple), atop sites (blue), weighted average of atop and 3-fold hollow (yellow), and experimentally measured value (green). **C**. Hydrogen surface coverage of 29.3 H/nm^2^.

Isopotential electron titration represents a new experimental tool to identify and measure charge transfer phenomena on catalyst surfaces, at catalytically relevant pressures and temperatures, in the absence of solvents or electrolytes interfaced with the adsorbing catalyst layer. While this work demonstrated the utility of IET for hydrogen adsorption on Pt, the technique is applicable to many combinations of materials and adsorbates of interest for chemical catalysis. Other Pt group metals, which may interact more favorably with other kinetically relevant adsorbates, are valuable consideration for future work. Molecules like carbon monoxide and ammonia are important to multiple catalytic chemistries of industrial relevance, and their strong adsorption on metal surfaces is likely to exhibit significant adsorbate–surface charge transfer that would be readily measurable through IET. While additional considerations such as back-donation and slow/irreversible desorption exist, IET remains applicable with minor methodology modifications. For example, for adsorbates that are not purely reversible in nature, adsorption measurements would be performed followed by increases in temperature to desorb surface species, as opposed to successive switches in gas phase chemical potential as demonstrated in this work. The applications of IET is only truly limited to surface interactions where a net charge transfer exists. The technique is also readily applicable to chemical reactions, facilitating the ability to measure the extent of charge transfer as adsorbates undergo thermochemical transformation on a surface. Complex reaction pathways serve as a potential challenge; however, this can be aided by computational and/or spectroscopic tools to predict which moieties are kinetically relevant or dominate surface coverage, deconvoluting the presence of several surface species and their contribution to measured extents of charge transfer. The correlation between change in work function, which scales with all catalytic parameters, and extent of charge transfer highlight both the importance and broad applicability of this technique. Beyond fundamental understanding alone, identifying adsorbate–surface interactions with significant extents of charge transfer allows for a quantitative method to identify field-responsive catalytic chemistries, and the surfaces on which they are most responsive to electric fields. Finally, recognizing the reversible nature of charge transfer, as opposed to a method of measurement, the approach described in this work provides a path to *apply* charge-voltage work to influence the rate of thermocatalytic reactions.

## 3 Conclusion

Isopotential electron titration was described as a technique to quantify the extent of charge transfer between adsorbed hydrogen and a nanoparticulate Pt film using a catalytic condenser architecture, where Pt was separated from a p^++^-Si electron sink by a high-k dielectric Hafnia insulator. Cycling between high and low surface hydrogen coverage under forced isopotential conditions between Pt and the p^++^-Si electron sink resulted in a flow of electrons originating from hydrogen adsorption, which was measured as a current between the Pt and Si layers. Integrating the current over time provided a direct measure of charge transfer between adsorbate and surface, where surface hydrogen was found to adopt a net partial positive charge as a result of electron donation to Pt. Across a temperature range of 125 −200 °C, representative of hydrogen surface coverages between 80 and 100%, a constant extent of charge transfer was measured (0.19 ± 0.01% |e|/H). The net direction and magnitude of charge transfer were found to be comparable with the theoretical predictions of a Bader charge analysis.

## 4 Materials and Methods

### 4.1 Catalytic Condenser Fabrication

Catalytic condensers were fabricated in a Class 100 cleanroom, with Pt (99.999%) as the catalyst layer, carbon as the conducting support, hafnia as the insulator, and p^++^-Si as the substrate. We have previously demonstrated that carbon serves as a conducting support that distributes charge across the catalytic layer.^47,48^ Hafnia was deposited over a degenerately p-doped (boron) Si wafer (Prime grade, 1–0–0, 4” diameter, 525 ± 25 μm thickness, ≤ 0.005 Ω-cm, single side polished, 2 SEMI flats) obtained from Waferpro (SKU C04005), using atomic layer deposition (ALD-150LE, Kurt J. Lesker), with alternating deposition of *tetrakis*(dimethylamido)hafnium (≥99.99, Sigma-Aldrich, 66610–25G) heated to 75 °C and deionized water. The Si wafer was held at 100 °C during five hundred deposition cycles, resulting in a HfO_2_ film of approximately (62 ± 2) nm as measured by ellipsometry (FS-1 Multi-Wavelength from Film-Sense). A shadow mask with 25 1 cm × 1 cm features was then placed onto the wafer and held onto the wafer using Kapton tape before a 3 nm carbon film was deposited by carbon thread evaporation (ACE600, Leica, using Leica carbon thread) using a base pressure of 10^–4^ mbar. Pt (ca. 1 nm) was deposited onto the masked C/HfO_2_/p^++^-Si using electron beam evaporation (SEC 600, CHA Industries). Finally, the Pt/C/HfO_2_/p^++^-Si wafer was cut into 25, 1 cm^2^ devices using a carbide tip scribe.

### 4.2 Catalytic Condenser Characterization

Selection criteria for devices used in this study entailed measurable values of capacitance (usually >100 nF/cm^2^) and high leakage resistance (>20 MΩ), consistent with our previous results for Pt/C/HfO_2_/p^++^-Si.^47–49^ Cyclic voltammetry (CV) was used to electronically characterize catalytic condensers, where capacitance was estimated as the average of current at 0 V normalized by sweep rate (0.5–1.5 V/s), while leakage resistance was taken to be the inverse of the slope of current versus voltage around 0 V for all sweep rates (Supporting Information, **Sec. S1B**). Typical room temperature values in air for the capacitance and leakage resistance were 220 nF/cm^2^ and 100 MΩ, respectively (Supporting Information, **Figure S3**). Characterization of catalytic condenser structures via electron microscopy was reported in our previous work.^49^

### 4.3 Electronic Reactor

Isopotential electron titration was carried out in two well-mixed, continuous-flow reactors at the University of Minnesota and the University of Houston. The flow rate of all gases was controlled using mass flow controllers (Brooks Instruments); effluent stream chemical composition was analyzed using an online gas chromatograph (7890B, Agilent) equipped with a thermal conductivity detector. Reactor temperature was controlled via a PID controller (CN7800, Omega) and a 1/16” type-K thermocouple (KQXL-116G-12, Omega) embedded in an electrical furnace (see Supporting Information, **Sec. S2C**). Device temperature was monitored using a second 1/16” type-K thermocouple (KQXL-116G-12, Omega) encased within a quartz sheath (4100N413, McMaster-Carr), with its tip vertically aligned near the center of the catalytic condenser. Flow diagrams and reactor details are provided in the Supporting Information (**Sections S.2–3)**. Catalytic condensers were placed on a custom-made in situ aluminum electronic stage (Grade 6061), with the p^++^-Si in direct contact with the stage, and a stainless-steel finger contacting the top Pt layer (Supporting Information, **Sec. S2E**). Stainless steel wires were used to connect each of the metal stage and finger to electrical feed throughs, with the Pt and p^++^-Si connected to the working and counter electrodes of a potentiostat (Squidstat Plus, Admiral Instruments), respectively.

### 4.4 In Situ Pretreatment

Once on the electronic stage, catalytic condensers were electronically characterized at room temperature under 50 sccm nitrogen (99.999%). While forcing a 0 V potential across a device (isopotential), the temperature was then linearly ramped (5 °C/min) to 200 °C where it was held for 30 min, after which device electronic properties were measured and compared against those at ambient conditions to confirm device integrity (capacitance and leakage resistance exceeded 100 nF/cm^2^ and 1 MΩ, respectively). In prior work, we observed a partial layer of oxide form over the Pt surface at room temperature via X-ray absorption spectroscopy.^51^ To ensure a purely metallic layer, the device was reduced at 200 °C for 30 min under a 50 sccm gaseous flow of hydrogen (99.999%); carbon-supported Pt is fully reduced by ∼80 °C.^65^ Additionally, 0.005 atm H_2_ was chosen as the hydrogen lean condition to avoid ppb O_2_ impurities in the ultrahigh purity nitrogen gas streams from reoxidizing the Pt surface. Finally, a device was allowed to achieve solid-state electrochemical equilibrium at the forced isopotential condition; this initial equilibration was critical for accurate measurements of adsorption currents free of extraneous electronic equilibration currents (defined as current of <10^–11^ A at 0 V). Additional details on this preconditioning are provided in the Supporting Information (**Sec. S4A**).

### 4.5 Isopotential Electronic Titration

IET measurements were performed following the equilibration period at 0 V potential difference between the Pt and p^++^-Si layers, holding at the desired adsorption temperature under 50 sccm molecular hydrogen (hydrogen rich) for 30 min to ensure a hydrogen covered Pt surface in electrochemical equilibrium with p^++^-Si. The gaseous environment was then instantaneously switched using a four-port valve (24UWE, Vici Valco) to a 0.5 mol % hydrogen environment (hydrogen lean), created by combining nitrogen and a 5% hydrogen in balance nitrogen (X02NI95C3000092, Airgas) that maintained a total gaseous flow rate of 50 sccm. Current flowing between Pt and p^++^-Si as a result of hydrogen desorption was recorded at a sampling frequency of 10 Hz by the potentiostat for 30 min, which was necessary to ensure the entirety of the transient current profile was captured (Supporting Information, **Sec. S4B–C**). Repeated cycles between hydrogen rich and lean environments at a single temperature (Figure 2A), allowed for the averaging of replicate measurements of adsorption/desorption induced charge transfer. The total pressure was maintained at 1 atm throughout all IET measurements. Detailed procedures for IET measurements and the corresponding data analysis are provided in the Supporting Information (**Sec. S4–5**). All reported errors were calculated at a 95% confidence level.

### 4.6 Density Functional Theory

DFT calculations were performed using the vienna ab initio simulation package (VASP)^66–71^ in conjunction with the atomic simulation environment (ASE)^72^ to determine the electronic structure and energy of hydrogen atoms adsorbed on Pt(111). The projected-augmented wave (PAW)^73^ method was employed to describe electron–ion interactions, with the Perdew–Burke–Ernzerhof (PBE)^74^ generalized gradient approximation (GGA)^75^ functional. A kinetic energy cutoff of 400 eV was used for the plane-wave basis set to solve the Kohn–Sham equations.

The Pt(111) surface was modeled as a four-layer slab in a (3 × 3) unit cell with periodic boundary conditions. The top two layers were relaxed while the bottom two layers fixed to their bulk positions. With the optimized lattice constant, the Pt site density in the model was 14.7 Pt/nm^2^, or 1.47 × 10^15^ atoms/cm^2^. The Brillouin zone was integrated using a (4 × 4 × 1) Monkhorst–Pack mesh.^76^ To account for molecules adsorbing on only one side of the slab, a dipole correction was applied. Geometries were optimized with a force convergence criterion of 0.05 eV/Å. Bader charge analysis was performed to estimate the partial electronic charge of atoms.^61,77,78^

### 4.7 Experimental Safety

Safety precautions were observed throughout the experiments, first through elimination and mitigation of hazards, then by engineering controls, and finally using personal protective equipment. During device fabrication, no unexpected hazards were encountered. The reactor was regularly leak-checked between experiments, and the reactor was kept at atmospheric pressure to minimize leaks and reactor vessel damage. Temperatures were set as low as reasonably possible to resolve the relationships described in the results, and the PID loop contained two 10 A fuses based on the maximum current rating of the heating apparatus to prevent overheating and short circuiting. Gas alarms were located in each room near the reactors to detect hydrogen at levels well below OSHA PELs. Potentiostats were grounded, with a maximum allowable current of one ampere.

## Supplementary Material



## Notes

Additional details about the device fabrication and testing (**Section S1**), the details of the University of Minnesota reactor system (**Section S2**), the details of the University of Houston reactor system (**Section S3**), the procedure used for IET experiments (**Section S4**), the raw data and subsequent analysis (**Section S5**), the H_2_ adsorption calculations (**Section S6**), a heat transfer model (**Section S7**), a reactor dynamics model to explain the current response (**Section S8**), and the electronic equilibrium analysis between Pt and p^++^-Si (**Section S9**) (PDF)
