# Supplementary material for: Isopotential Electron Titration: Hydrogen Adsorbate-Metal Charge Transfer
Source: ACS Cent Sci. 2025 Sep 15;11(11):2063–73. doi: 10.1021/acscentsci.5c00851 (PMC12670304; doi:10.1021/acscentsci.5c00851)
Supplement: Supplementary file 1 [file oc5c00851_si_001.pdf]

SUPPORTING INFORMATION

Isopotential Electron Titration: Hydrogen Adsorbate-Metal Charge Transfer

Justin A. Hopkins[1,2,‡], Benjamin J. Page[1,3,‡], Shengguang Wang[1,3,4], Jesse R. Canavan[1,2], Jason A. Chalmers[1,5], Susannah L. Scott[1,5], Lars C. Grabow[1,3,4], James R. McKone[6], Paul J. Dauenhauer[1,2], Omar A. Abdelrahman[1,3,*]

[1] Center for Programmable Energy Catalysis, University of Minnesota, Department of Chemical Engineering & Materials Science, 421 Washington Ave. SE, Minneapolis, MN, USA, 55455, cpec.umn.edu
[2] Department of Chemical Engineering & Materials Science, University of Minnesota, 421 Washington Ave. SE, Minneapolis, MN, USA, 55455
[3] William A. Brookshire Department of Chemical and Biomolecular Engineering, University of Houston, 4226 Martin Luther King Blvd., Houston, TX, USA 77204
[4] Texas Center for Superconductivity, University of Houston, 4226 Martin Luther King Blvd., Houston, TX, USA 77204
[5] Department of Chemical Engineering, University of California, Santa Barbara, CA, 93106
[6] Department of Chemical and Petroleum Engineering, University of Pittsburgh, 3700 O'Hara St, Pittsburgh, PA, USA, 15213

* Corresponding author: oabdel@uh.edu

‡ These Authors contributed equally

Table of Contents

S1. Condenser Fabrication and Testing....3
A. Device Fabrication....3
B. Electronic Property Measurements....4
S2. University Minnesota Reactor System....6
A. Reactor Design....6
B. Reactor Vessel....8
C. Reactor Temperature Control....8
D. Reactor Residence Time Distribution....9
E. In Situ Electrical Testing Stage....10
S3. University of Houston Reactor System....11
A. Reactor Design....11
B. Reactor Vessel....13
C. Reactor Temperature Control....14

D. In Situ Electrical Testing Stage 14
E. Potentiostat Parameters 15
S4. Isopotential Electron Titrations – Procedure 16
S5. Raw Data and Data Analysis 19
A. Electrical Properties During Heating 19
B. IET Raw Data Analysis Methods 20
C. IET Charge Integration Data Tables 21
D. Titration Control Experiments 23
E. Device Stability and Failure Modes 29
S6. Hydrogen Adsorption 31
A. Hydrogen Coverage 31
B. Main Reference for Adsorption Values 31
C. Work Function Change of Pt upon H2 Adsorption 33
D. Sensitivity to $\underline{\Delta H}$ and $\underline{\Delta S}$ 34
S7. Heat Transfer Model 40
S8. Reactor Dynamics Model to Explain Current Response 42
S9. Pt and Si Charge Equilibration Analysis 44
References 45

S1. Condenser Fabrication and Testing

A. *Device Fabrication*

The catalytic condensers used throughout these studies were fabricated at the University of Minnesota and had an architecture of Pt/C/HfO_2/p^{++}-Si with an exposed Pt surface area of 1 cm^2. Condenser fabrication was consistent with our previous works, and additional fabrication and characterization details for the various materials can be found there.[1–4] Briefly, a 4" p^{++}-Si wafer (supplied from Wafer pro, 0.5 mm thick, resistivity of 0.005 Ω cm) was obtained and cleaned with an isopropanol, methanol, and acetone rinse followed by drying under N_2 flow. The native silicon oxide was not removed for these fabrications.

The hafnia dielectric film was deposited over the entire 4" wafer using atomic layer deposition (ALD, Kurt J. Lesker ALD-150LE). The precursor used was tetrakis(dimethylamido)hafnium (IV) (TDMAH, stored at 75 ^oC), and the co-reactant was water vapor. Prior to deposition, the Si wafer was allowed to equilibrate to a temperature of 100 ^oC. A total of 500 cycles of TDMAH + H_2O was used to obtain a final film thickness between 60 and 70 nm (growth per cycle of approximately 1.24 Å/cycle).

The HfO_2/p^{++}-Si film was then rinsed again with isopropanol, methanol, and acetone prior to placing a shadow mask of twenty-five 1 cm x 1 cm cutouts, and the mask was adhered to the wafer using double-side Kapton tape. A carbon film was then deposited by carbon thread evaporation (Leica ACE600) at a base pressure of 10^{-4} mbar such that twenty-five, 1 cm^2 devices were fabricated. For Device #1 only, the carbon film was instead sputtered using an AJA ATC 2000 sputtering system with a pyrolytic carbon target, 5 mTorr of Ar (20 sccm), and a sputtering power of 250 W. The total sputtering time was 300 seconds for every 1 nm of carbon deposited, and films of 1 nm and 3 nm carbon were used throughout the study. The specific purpose of the carbon layer was to provide lateral conductivity to the Pt layer.

The mask remained adhered to the wafer for the Pt deposition using an electron beam evaporator (CHA model SEC 600). Approximately 1 nm of Pt (monitored using a quartz crystal microbalance in the deposition chamber) was deposited onto the device at a rate of 1 Å/s. After Pt deposition, the mask was removed from the wafer. The twenty-five 1 cm^2 Pt/C/HfO_2/Si devices were then cleaved using a carbide tipped pen by lightly drawing a line where the wafer should be cut. Following this, each device received a unique ID number and electronic testing was conducted. A cartoon of these condensers is shown in **Figure S1A** with a picture of an actual device shown in **Figure S1B** below. Representative electron microscopy, X-ray diffraction, and atomic force microscopy characterization are presented in previous fabrication-centered work.[1–3]

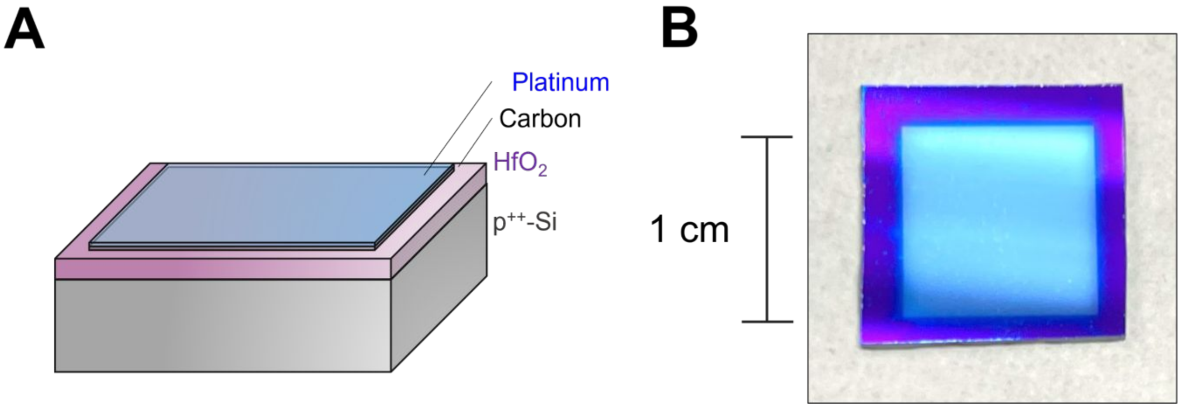

Figure S1: **(A)** A cartoon of the catalytic condenser's layers showing p^{++}-Si in gray, HfO_2 in purple, carbon in gray (under the Pt), and Pt in blue. **(B)** A top view photograph of a catalytic condenser showing the HfO_2 dielectric (purple color) and the Pt/C layer (blue color).

B. Electronic Property Measurements

Cyclic voltammetry (CV) experiments were conducted on the 1 cm^2 Pt/C/HfO_2/Si devices to estimate capacitance and resistance values of the overall device. The working electrodes of a potentiostat were always attached to the top Pt/C through a stainless-steel finger while the counter electrode was attached to the p^{++}-Si through a conductive stage (**Figure S2**). This contact scheme is described further in **Section S2E.**

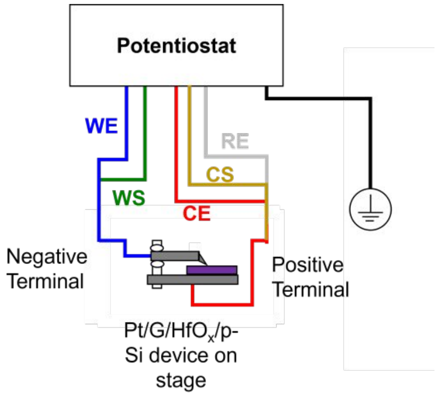

Figure S2: Wiring scheme for the potentiostat and devices showing the working electrode (blue) and working sense electrode (green) contacting the Pt/C side of the device; the counter electrode (red), counter sense electrode (gold), and reference electrode (gray) contacting the Si side of the stage.

Current versus voltage values were measured at linear voltage sweep rates ranging from 0.50 to 1.50 V/s were used. The capacitance of a device was calculated by fitting the current (I) at 0 V versus the voltage sweep rate (dV/dt) considering a charge balance with constant capacitance,

$$I(0) = C\frac{dV}{dt} \tag{S1}$$

Typical capacitance values for a Pt/C/60-70 nm HfO_2/Si device was 200 nF/cm^2. Device resistance was also estimated using current vs. voltage data. Specifically, the resistance (R) was calculated as the inverse of the slope of the current versus voltage data around 0 V,

$$R \approx \left(\frac{dI}{dV}\right)^{-1}\Bigg|_{V=0} \tag{S2}$$

Resistance values varied based on the temperature tested, consistent with our previous reports.[2] At room temperature, a typical resistance value was ~200 MΩ. More charge carriers can jump the energetic barrier between the Pt and/or Si Fermi level with increasing temperature and the conduction band of the HfO_2. The resistance decreased to about 2 MΩ at 200 ^{o}C. The resistance measured accounts for both the in-series resistance (i.e., the wires, Pt/C, and Si) summed with the resistance of HfO_2.

For isopotential electron titration experiments, the most important device characteristic is believed to be the device resistance. In measuring charge transfer between the Pt and Si, it is important to measure all of the transfer current as a result of adsorption/desorption events. If the electrical resistance of the HfO_2 is much greater than that of the in-series resistance (e.g., potentiostat wires, Pt/C, and Si), then the overwhelming majority of the equilibrating current must flow through the potentiostat where it can be measured. **Figure S3A** shows an example CV with typical capacitance and resistances at room temperature in which the resistance of the HfO_2 is much greater than the in-series resistance. If the resistance of the HfO_2 is comparable to the in-series resistance, then charge will leak through the HfO_2 as fast as it can be injected into the Pt/C and Si layers. **Figure S3B** shows a CV in which the HfO_2 dielectric film was intentionally shorted by a scratch across the Pt/C layer that was visible to the eye, where minimal hysteresis is observed, indicating that charge cannot be stored and that the in-series resistance is comparable to/greater than that of the HfO_2 dielectric.

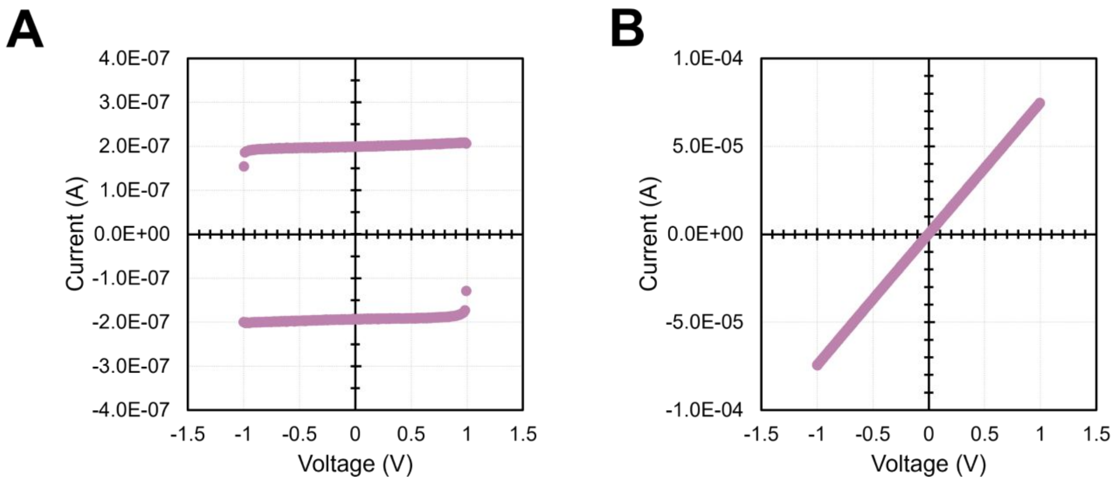

Figure S3: **(A)** Current versus voltage for a device with capacitance of 199 nF/cm^2 and resistance of 131 MΩ, 1V/s sweep rate. The positive current represents the positive sweeps while negative currents represented the negative sweep. The hysteresis between the positive and negative sweeps indicated the HfO_2 dielectric is significantly more resistive than the in-series resistance **(B)** Current versus voltage for a device in which the HfO_2 dielectric film was shorted, 1V/s sweep rate.

S2. University Minnesota Reactor System

A. Reactor Design

A flow reactor system was used during these isopotential electron titration experiments. The system was equipped with four mass flow controllers (MFCs, Brooks): two for calibrated for 99.999% N_2, one calibrated for 99.999% H_2, and one calibrated for 5% H_2 in N_2. The four MFCs were split into two streams such that each H_2 stream was combined with a N_2 MFC. These streams were connected to a four-port switching valve (Vici Valco) that connected either stream to either the reactor inlet or an auxiliary vent. The outlet of the reactor was attached to two three-port valves which allowed for the reactor effluent to be routed either to a bellows pump (Senior Metal Bellows) or bypassing pump. The effluent stream was connected to a six-port valve equipped with a 1 ml gas sampling loop to be analyzed by a gas chromatograph. The effluent from this six-port valve was then routed to another vent. A process flow diagram of this reactor system is shown in **Figure S4**. 1/8" stainless steel tubing was used to connect all units of the reactor (with the exception of the tubing around the six-port valve which was 1/16" stainless steel to fit under an Agilent heated valve box). Pressure gauges were placed around the reactor vessel and pump to ensure that over pressurization did not occur. A photograph of the reactor is shown in **Figure S5**. For electrical measurements, a SquidStat Pro from Admiral Instruments was used.

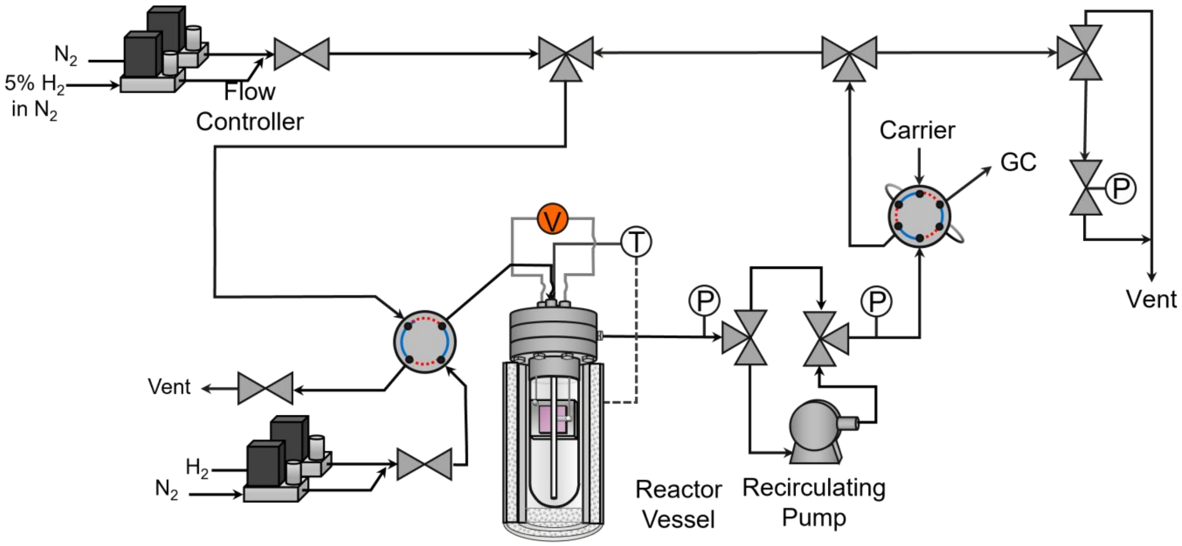

Figure S4: the reactor process flow diagram of the reactor system used in this study.

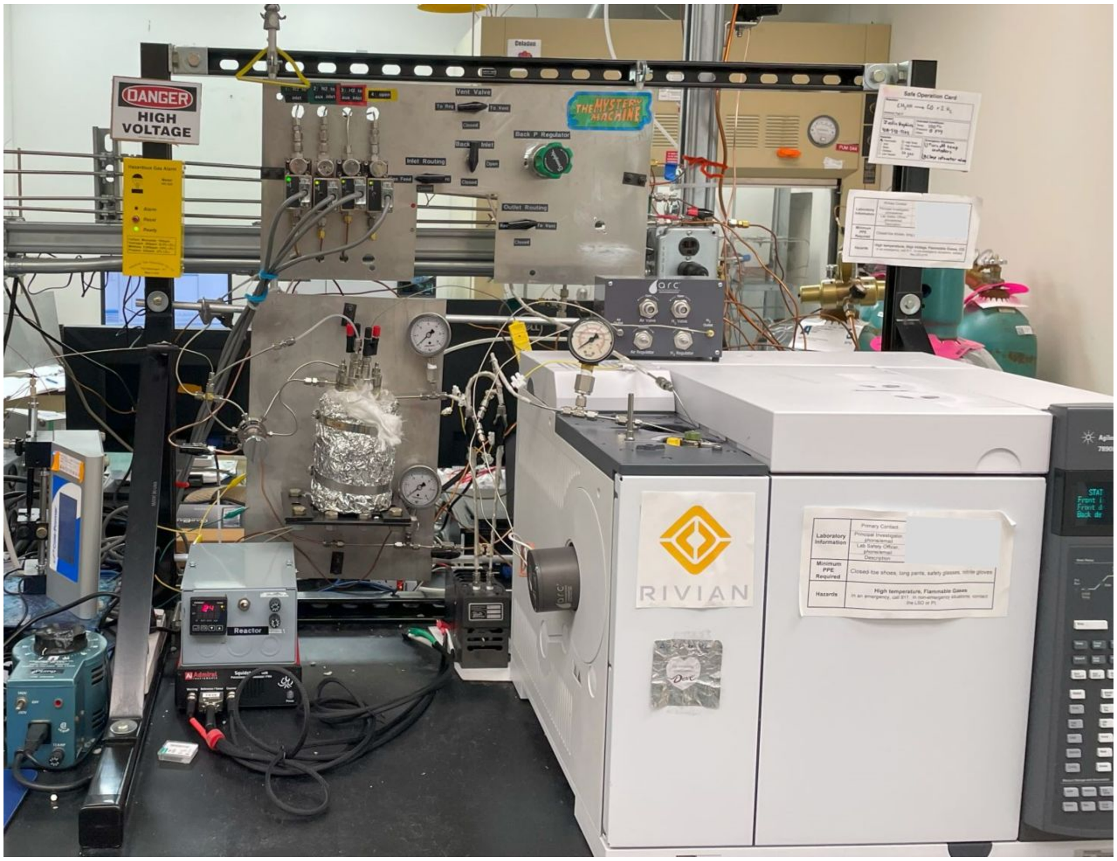

Figure S5: a photograph of the reactor in operation.

B. *Reactor Vessel*

The reactor vessel itself consisted of a 2.75" CF feedthrough (two feedthroughs) flange modified to insert a K type thermocouple and a glass inlet tube, a 2.75" CF double-side flange modified with an outlet tube, and a glass-to-metal flange adapter in which the glass tube was closed at the bottom end. The glass inlet tube injects gases from into the bottom of the reactor and the outlet tube is located at the top such that this reactor operates up flow. The reactor vessel was approximately 160 mL in total.

C. *Reactor Temperature Control*

A K type thermocouple was inserted into the reactor through the feedthrough flange such that the tip of the thermocouple was about halfway down the reactor tube. The thermocouple then connected to an Omega CN7800 temperature control unit. The output of the controller activated a solid-state relay which would allow current to pass through resistive heating tape to heat the reactor. These electrical circuits had a 10 A fuse located on them in the case of a short circuit in the system.

Care was taken to ensure that the setpoint temperature in these experiments matched the temperature the device experienced as closely as possible. Since the reactor vessel is 160-mL and primarily consists of glass, a custom heater was designed that ensured isothermal conditions for over half of the reactor tube. The heater consisted of: (1) a 2.25" O.D. and 0.25" wall thickness aluminum tube which fit loosely around the glass tube, (2) 4 feet long resistive heating tape wrapped around the aluminum tube, (3) quartz insulation to a total O.D. of about 4", (4) a 304 stainless steel sheet of 0.0070" thick wrapped around the insulation, and (5) aluminum foil to contain any loose insulation. All of the various layers were secured together with hose clamps. The vertical cross section of the heater with the reactor vessel is shown in **Figure S6A**, the areal cross section of the heater is shown in **Figure S6B**, and a photograph of the heater with the reactor tube in it is shown in **Figure S6C**. To measure the temperature gradient along the length axis of the reactor, the control thermocouple was held midway through the reactor tube and a second K type thermocouple was slid along the axis through the glass inlet tube hole, making sure to let the measured temperature reach steady state at each location before recording the measurement. **Figure S6D** shows the measured temperature along the axis of the reactor tube with the 0 cm location being the tip of the glass tube (i.e., bottom of the reactor) and the flange of the metal-to-flange adapter being located at 11 cm. From these results, the temperature within the bottom 7 cm of the reactor was within 200 $\pm$ 2 ^{o}C when the setpoint of the PID was 200 ^{o}C. Therefore, the electrical stage and device was positioned within this isothermal zone of the reactor.

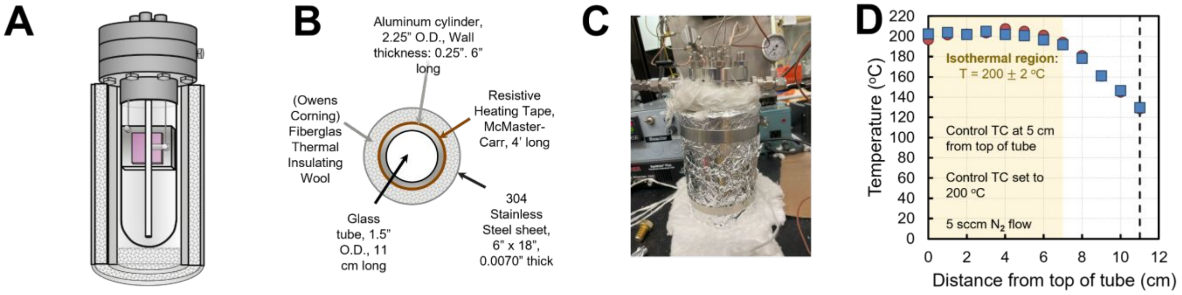

Figure S6: **(A)** a schematic of the cross section of the heater indicating that the custom heater envelops the reactor glass tube and sits flush with the flanges of the reactor vessel, **(B)** a cross sectional schematic of the reactor showing that the heater consists of an aluminum cylinder, resistive heat tape, fiberglass insulation, and a stainless steel wrapped sheet, **(C)** a picture of the heater**, (D)** a graph of the temperature distribution of the custom heater emphasizing that 7 cm from the tip of the tube has a temperature profile of 200 $\pm$ 2 ^{o}C when the setpoint is set to 200 ^{o}C. Note that the temperature profile shows a drop-off towards the end of the reactor vessel since the stainless-steel flanges are not heated.

D. *Reactor Residence Time Distribution*

A residence time distribution experiment was performed on the reactor to determine its volume and effective hydrodynamics. Specifically, the reactor was set at 200 ^{o}C and 2 sccm of ultrahigh purity N_2 was flowed. A short GC run was used to measure the reactor effluent's H_2 concentration every 5 minutes. Confirming that there was no H_2 in the effluent, the gas flow was switched to 150 sccm H_2 for 1 second to introduce a pulse tracer. This was done 3 minutes into a GC trial in an attempt to catch any time lag in H_2 arriving to the GC. The peak area of H_2 (A) measured by the GC-TCD was then used to calculate a residence time distribution function (E) following **Equation S3** and the residence time from **Equation S4**:[5]

$$E(t) = \frac{A(t)}{\int_0^\infty A(t)dt} \quad \text{(S3)}$$

$$\tau = \frac{V}{Q} = \int_0^\infty tE(t)dt \quad \text{(S4)}$$

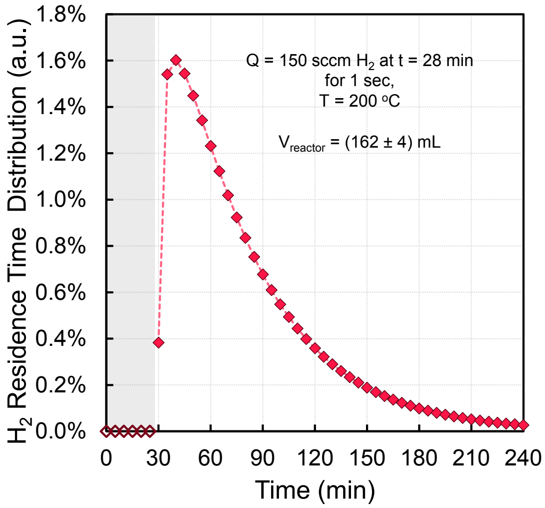

Figure S7: The residence time distribution function vs. time for the U Minnesota reactor at 200 ^{o}C with 2 sccm N_2 flow. No H_2 was flowed from t = 0 to t = 28 min. At t = 28 min, 150 sccm H_2 was flowed for 1 second to introduce a pulse tracer. The red dashed line is meant to guide the eye.

From **Figure S7**, the residence time distribution function versus time shows us that there is a small time lag for the H_2 to reach the GC followed by an exponential decay. Thus, the reactor can be modeled as a small volume plug flow reactor with a significant portion of its volume acting as a well-mixed reactor. The residence time calculated from **Equation S4** was 51 ± 1 min. Knowing that 2 sccm of N_2 was constantly flowing (and acknowledging that the flowrate in mL/min is larger than 2 sccm since the temperature is greater than the standard temperature), this indicates that the reactor volume is about 162 ± 4 mL with >75% of that volume acting as a well-mixed reactor. Using this volume, we can determine the residence for the reactor during IET experiments where we used a flowrate of 50 sccm instead of 2 sccm. Since the flowrate was 25x higher during IET experiments than during this RTD experiment, the residence time during IET was only 2 min. Therefore, performing gas switches for IET every 30-minutes would correspond to 15 residence times and the reactor should be at steady state in composition.

E. *In Situ Electrical Testing Stage*

In-situ electrical measurements in the environmentally controlled volume were performed using an electrical stage (**Figure S8**). The stage consisted of a 1.25 cm x 1.25 cm x 0.5 cm aluminum body with screw holes tapped in various places on the stage. A metallic screw is inserted into the top left corner of the stage and a stainless-steel wire (22 gauge) was wrapped around the screw to make the Si contact. A ceramic screw was inserted into the middle right of the stage with a stainless-steel finger contact and ceramic washer

underneath to prevent shorting the electrical stage. Another stainless-steel wire was wrapped around the ceramic screw, and a ceramic washer is placed on top of the wire coil. Screwing the ceramic washer into the stage places compression on the wire coil which in turn compresses the finger contact onto the Pt/C layer. Over tightening the ceramic screw could result in scratches to the Pt/C/HfO_2 layers, which could short circuit the device (**Figure S3B**). The wires were cut long enough such that the stage and device sat in the isothermal zone of the reactor at the same vertical height as the thermocouple.

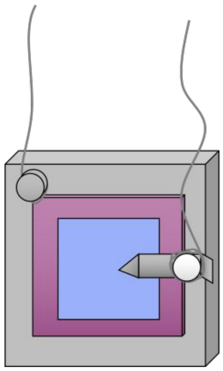

Figure S8: Electrical testing stage diagram showing the aluminum stage, aluminum conducting screw on the top left, ceramic screw in the middle right, and stainless-steel finger in the middle right under the ceramic screw. A cartoon of a condenser is shown to demonstrate how a device is held on this stage.

S3. University of Houston Reactor System

A. Reactor Design

A Continuously Stirred-Tank Reactor (CSTR) was built for the purpose of analyzing electronic and catalytic properties of catalytic condensers. Bypass and reactor streams were constructed using electronic mass flow controllers (MFCs, Brooks: 5850S) with 1/8" stainless steel tubing. A 1/8" 'ultra-wide' 6-port valve with a type 'E' rotor and 2" standoff (Vici Valco: 26UWE) was used with a pressurized actuator to rapidly switch between bypass and reactor streams. A pressure gauge was attached to leak-test the reactor. A complete diagram and parts list can be found in **Figure S9** and **Table S1**. While differences in the mechanics of operating control parameters existed between the two reactors, the chemical potential of the gas and the electronics within the reactors were the same, making them functionally identical.

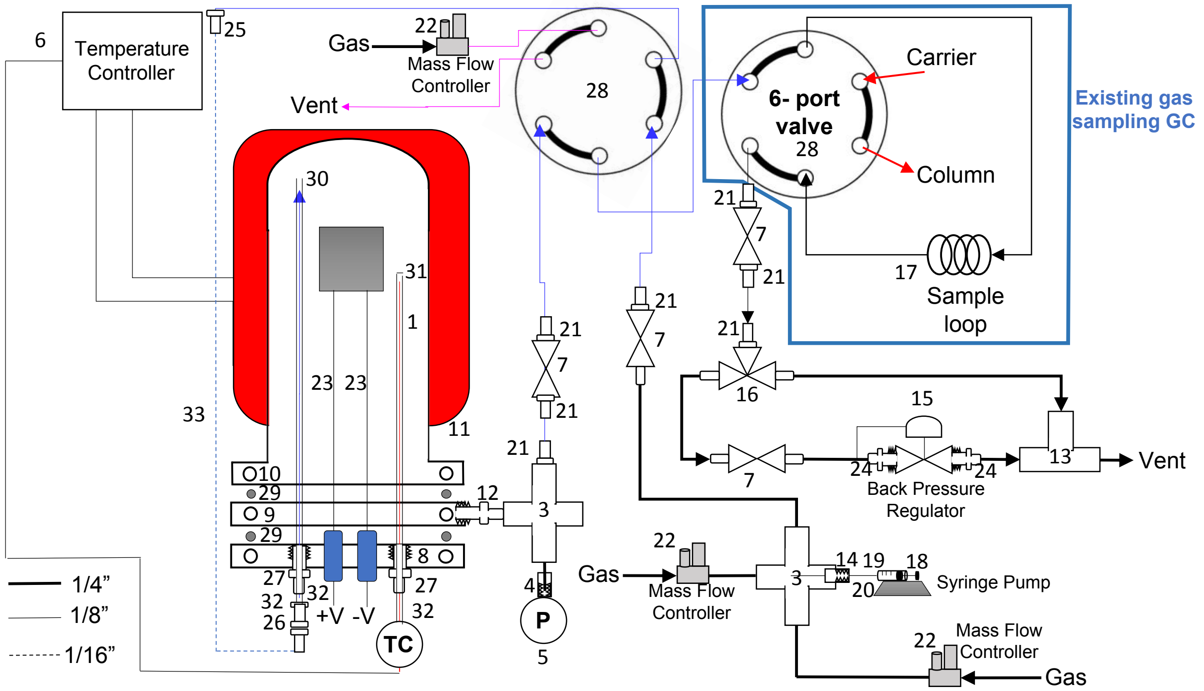

Figure S9: Reactor diagram used at University of Houston to measure extent of charge transfer and controls on Pt catalytic condenser #2.

Table S1: Parts list of the reactor used at University of Houston to measure extent of charge transfer and controls on Pt catalytic condenser #2.

Item	Vendor	Description	Part Number	Quantity
1	Omega	High Temp Low Drift TC Probes with Standard Size Connectors type-K	KQXL-116G-12	2
2	Love Controls	Temperature Limiter	TSF-4011	1
3	Swagelok	Union Cross, 1/4 in. Tube OD	SS-400-4	2
4	Swagelok	Female Connector, 1/4 in. Tube OD x 1/4 in. Female NPT	SS-400-7-4	1
5	McMaster	Pressure Gauge, 1/4 NPT Male Bottom Connection, 2-1/2" Dial, 0-15psi	3795K13	1
6	Omega	Temperature Controller	CN7800	1
7	Swagelok	1-Piece 40G Series Ball Valve, 0.6 Cv, 1/4 in. Swagelok Tube Fitting	SS-42GS4	4
8	Accu-Glass	MHV - Single Ended, Grounded Shield Feedthrough x2 on a 2.75" CF Flange	MHV-GS2-275	1
9	LDS Vacuum	2.75" OD Double Sided CF Flange with two 1/8" NPT Holes	275DxSP12-2	1
10	Accu-glass	Adapter, Stainless steel to Pyrex glass, 1.5 inch, on 2.75 inch ConFlat flange	SPD-150-275	1
11	ThermCraft	Aluminum Block Furnace, Described in S2.C of The SI	VF-360-1.5-6-S	1
12	Swagelok	Bored-Through Male Connector, 1/4 in. Tube OD x 1/8 in. Male NPT	SS-400-1-2BT	2
13	Swagelok	Union Tee, 1/4 in. Tube OD	SS-400-3	1
14	Vici Valco	1/16" Bore Tube Adapter – 1.8" Length	ZLTA41	1
15	Swagelok	BP Regulator, 0 to 50 psig (3.4 bar), A Configuration, FKM Seat, 1/4 in. FNPT, 0.20 Cv	KBP1E0A4A5A20000	1
16	Swagelok	1-Piece 40G Series 3-Way Ball Valve, 0.35 Cv, 1/4 in. Swagelok Tube Fitting	SS-42GXS4	1
17	Vici Valco	1/16", 0.75 mm port, 2ml sample loop	SL2KCUW	1
18	Hamilton	500 µL Gastight Syringe Model 1750 TLL, PTFE Luer Lock	81220	1
19	Idex	Threaded Luer Adapter, Natural PEEK, 0.050" Bore, Female Luer x Female 10-32 Coned	P-659	1
20	Idex	Fingertight Two-Piece Fitting, Standard Knurl, Natural PEEK, 1/16" OD Tubing, 10-32 Coned	F-330	1
21	Swagelok	1/8 in. Tube OD x 1/4 in. Swagelok Tube Adapter	SS-200-R-4	7
22	Brooks	Mass flow controller, 300 sccm maximum rating	5850S	3
23	McMaster	Tantalum Wire, 0.012" Diameter, 5 Feet	5627N22	1
24	Swagelok	Male Tube Adapter, 1/4 in. Tube OD x 1/4 in. Male NPT	SS-4-TA-1-4	2
25	Swagelok	Reducer, 1/16 in. Tube OD male x 1/8 in. male Swagelok Tube Adapter	SS-200-6-1	1
26	Swagelok	Reducer, 1/16 in. male Tube OD x 1/8 in. female Swagelok Tube Adapter	SS-100-R-2	1
27	Swagelok	Bored-Through Male Connector, 1/8 in. Tube OD x 1/8 in. Male NPT	SS-200-1-2BT	2
28	Vici Valco	1/8" 6-port Switching Valve (type 'E' rotor) w/ 2" standoff	26UWE	2
29	Kurt Lesker	2.75" OD, 1.45" ID Copper Crush Washers (pack of 10)	DN35CF-DN40CF	1
30	Technical Glass Products	3mm OD Quartz Tubing	1X3	1
31	Technical Glass Products	3mm OD Quartz Tubing – 1 Side Domed Off (in-house)	1X3	1
32	Restek	1/8" Graphite Ferrules (pack of 10)	20208	3
33	Vici Valco	1/16" OD PEEK tubing 0.005" Tubing ID	TPK105-10FT	1

B. <u>Reactor Vessel</u>

A 1.5" diameter domed Pyrex tube glass adapter (Accu-Glass: SPD-150-275) was used to house the catalytic condenser. An open-ended 3mm OD quartz tubing (Tech. Glass Products: 1x3) was used as a reactor inlet and a close-ended sheath was used to house the type-K thermocouple (Omega: KQXL-116G-

12) inside the reactor without interacting with the surrounding chemical(s). Both quartz tubes, as well as two electrical feedthroughs were installed on a grounded shield feedthrough x2 2.75” CF flange (Accu-Glass: MHV-GS2-275) with custom porting for the quartz feedthroughs.

The catalytic condenser was supported on a stage using a stainless-steel cantilever to contact the catalyst layer. The cantilever was supported by a ceramic screw and washer to electrically insulate the cantilever from the stage.

C. Reactor Temperature Control

The reactor was heated using a custom-built furnace to provide an isothermal zone across the entire reactor vessel. **Figure S6** shows construction and testing of furnace. The furnace had two type-K thermocouples to prevent overheating of the reactor. One thermocouple was inserted into the quartz sheath inside the reactor vessel, which was attached to a temperature limiter (Love Controls: TSF-4011). The second thermocouple was inserted between the aluminum block and the heating tape of the furnace.

D. In Situ Electrical Testing Stage

Prior to each experiment, catalytic condensers were placed on an electronic stage, housed within the heated zone of the reactor and characterized by in-situ cyclic voltammetry to ensure the ability to condense charge. Typically, a capacitance >100 nF/cm^2 at 300 K and 1 atm N_2 was measured for the Pt condensers in this work as shown in **Figure S10**.

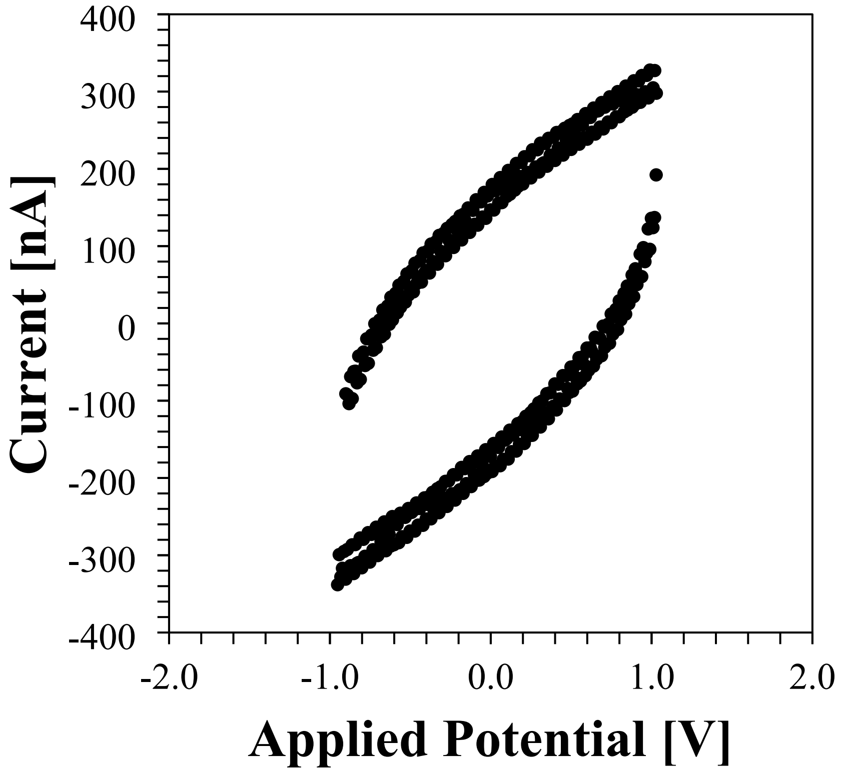

Figure S10: Cyclic voltammogram (1000 mV/s) of Pt catalytic condenser #2 in 1 atm nitrogen (50 sccm) at 473 K. Measured capacitance of 147 nF/cm^2.

E. Potentiostat Parameters

Data was collected using a potentiostat (Gamry: 1010e) using the settings described below in **Table S2** and **Table S3**. Once data was collected from the experiment, a baseline for each adsorption/desorption event was calculated by averaging the current value between two defined points on the curve once the device had returned to electrochemical equilibrium. The current profile and baseline profile were integrated. The difference between the current profile and the baseline profile was the charge transferred as a function of hydrogen adsorption/desorption. The absolute value of the adsorption/desorption events were averaged to calculate the charge transfer at that temperature setpoint. The adsorption/desorption charges were averaged because the amount of hydrogen adsorbed is equal to the amount of hydrogen desorbed, meaning the respective charge transfers are equal and opposite. The first adsorption/desorption cycle was not included in the average because the first adsorption curve typically served to reduce the Pt layer.

Table S2: Gamry 1010e hardware settings used to collect experimental data.

Parameter	Setting
Potentiostat	IFC1010-24136
I/E AutoRange	ON
Ich AutoRange	ON
Ich Filter	1 KHz
Sense Cable ID	R600 Shielded Cell Cable - 60cm
Framework Version	7.8.1
Instrument Version	4.4.0
Pstat Model	Interface 1010

Table S3: Gamry 1010e experimental settings used to collect data.

Parameter	Setting
Initial E (V)	0 vs. E_{OC}
Initial Time (s)	8000
Final E (V)	0 vs. E_{OC}
Final Time (s)	8000
Sample Period (s)	0.1
Limit I (mA/cm^2)	10
Conditioning Time (s)	15
Conditioning E (V)	0
Init. Delay Time (s)	5
Init. Delay Stab. (mV/s)	0

S4. Isopotential Electron Titrations – Procedure

A. Initial Setup

If a catalytic condenser was present, the vessel was cooled to room temperature in 15 psia N_2 at 50 standard cubic centimeters per minute (sccm). The N_2 stream was shut off, and 1/8" gas inlet and outlet compression fittings were unscrewed and removed from the vessel, which was then removed from the heater. To open the vessel, using gloves, six nuts and bolts on the Conflat (CF) flange assembly were unscrewed in a star pattern using 45° turn increments until they could be loosened by hand. Once open, the grounded shield feedthrough flange (**Item #8, Table S1**) holding the aluminum stage and catalytic condenser was separated from the other two flanges (**Items #9, #10, Table S1**). Both working and counter electrode wires were disconnected from the electrical feedthroughs on the grounded shield feedthrough flange. The aluminum stage and wires were placed on a kimwipe with the catalytic condenser face up. The

catalytic condenser was removed from the aluminum stage by holding the cantilever still using a set of tweezers while loosening the ceramic screw on the aluminum stage until the cantilever could freely move off the catalytic condenser without touching the surface. The catalytic condenser was removed by grabbing the sides with a plastic set of tweezers. A new catalytic condenser was placed in the center of the electrical stage using the same cleaned plastic set of tweezers with the catalyst layer face up, free of direct contact with the conducting screw. The cavalier was positioned above the center of the platinum layer and fixed in place with a set of tweezers. The ceramic screw was tightened while the catalytic condenser was held in place until the cantilever and catalytic condenser were in contact and just barely immobile (**Figure S8**). Working and counter electrode wires were reattached to the electrical feedthroughs on the grounded shield feedthrough flange by connecting the wire in contact with the cantilever to the feedthrough in contact with the potentiostat's working electrode, and the wire in contact with the aluminum stage (p-type silicon) to the feedthrough in contact with the potentiostat's counter electrode. A multimeter was used to make sure the aluminum stage and wires were not in contact with the grounded shield feedthrough flange. Once confirmed, a cyclic voltammogram was measured at 1.00 V/s between -1.00 V and 1.00 V vs V_{OC} to confirm the catalytic condenser was in electrical contact with the potentiostat and could condense charge. To reassemble the vessel, replace the crush gaskets between the CF flanges, then align the grounded shield feedthrough flange with the double side flange and metal-to-glass flange adapter, and screw the flanges together using the six nuts and bolts in a star pattern with 45° turns until tight. The vessel was reinserted into the heater, and the inlet and outlet compression fittings were reconnected. Once assembled, the vessel was leak tested. At the University of Minnesota, a bellows pump was used to pull a light vacuum on the vessel by closing a three-way pump valve to seal the vessel outlet from the vent where the pressure did not change. At the University of Houston, the assembled vessel was pressurized at 20 ^oC with 20 psia nitrogen to confirm the absence of any measurable leak over 15 minutes. A multimeter was again used to check if the catalytic condenser was in electrical contact with the vessel by placing one terminal on the working electrode wire, and the other terminal on the outer vessel walls. Once confirmed, a second cyclic voltammogram was measured using the same settings to confirm inserting the catalytic condenser into the reactor did not cause any increase in leak current or decrease in capacitance. Finally, the vessel was purged with 15 psia N_2 at 100 sccm for 10 minutes, then the flowrate was decreased to 15 psia N_2 at 50 sccm, and the temperature setpoint was adjusted to 200 ^oC with a ramp of 3 ^oC/min.

Once the temperature reached steady state, the catalyst was reduced by flowing 15 psia H_2 at 50 sccm for 30 minutes, then purged with 15 psia N_2 at 50 sccm. Another cyclic voltammogram was measured using the aforementioned settings to confirm heating and reducing the catalytic condenser did not dimmish its ability to store charge. To reach electrochemical equilibrium, 0 V vs V_{OC} was applied for 12 hours until

di/dt = 0 pA. Alternatively, -5 V vs V_{OC} was applied for 5-minute intervals to eliminate the current baseline, as discussed in **Section S5A**.

B. Isopotential Electron Titration Procedure: Adsorption and Desorption Cycle Experiments

The temperature was adjusted to the desired setpoint and reached steady state while flowing 15 psia of N_2 at 50 sccm. A final cyclic voltammogram was measured using the same parameters to confirm that the catalytic condenser stored charge before the start of the experiment. Then, 15 psia of H_2 at 50 sccm was introduced to the system while running a 0 V chronoamperometry experiment on the potentiostat to ensure the platinum surface was reduced and coated with H* to begin the trial, sampling the current every 10 seconds during this period. Once the device had reached electrochemical equilibrium, as indicated by no change in current with respect to time, a flow of 5 sccm of 5% H_2 and 45 sccm of N_2 (resulting in a total of 15 psia of 0.5% H_2 at 50 sccm) was introduced while actively measuring current. The sampling rate was changed to 0.05 s for the first minute, 1 s for the second minute, and back to every 10 s for the remaining 28 minutes. After 30 minutes, the stream was switched to 15 psia of 99.999% H_2 at 50 sccm while maintaining the same sampling frequency scheme and confirm that the vessel reached steady state with a gas chromatograph (GC). Finally, the cycle was repeated once more at the same temperature.

C. Isopotential Electron Titration Procedure: Adsorption and Reset Experiments

The temperature was adjusted to the desired setpoint while flowing 15 psia of N_2 at 50 sccm which allowed the temperature to reach steady state. A cyclic voltammogram was measured using the aforementioned settings to confirm the catalytic condenser stored charge. 15 psia of H_2 at 50 sccm was introduced while holding a 0 V drop across the catalytic condenser to ensure the platinum surface was coated with H* to start the trial, sampling the current every 10 seconds. 15 psia of 0.5% H_2 at 50 sccm was introduced to replace the gas environment, while a bellows bump was used to decrease the vessel pressure to 500 Torr, which was sufficient to reset the device surface consistently. After the desorption peak was measured over the course of 3 minutes, the pump was turned off and the downstream valve was closed to prevent backfilling. The stream of 0.5% H_2 was used until the pressure across the vessel returned to 15 psia, where the downstream valve was opened. The catalytic condenser was given 10 seconds to equilibrate with 0.5% H_2, then sampled every 0.1 seconds at which point the stream was switched to 15 psia of H_2 at 50 sccm using the four-port switching valve. After 1 minute, the sampling rate was reduced to every 1 second. After 10 minutes, the stream was switched back to 0.5% H_2 flow to reset the gas phase for further trials. A cyclic voltammogram was measured every 3 trials at a given temperature or every time the temperature setpoint changed to ensure the catalytic condenser could still store charge.

S5. Raw Data and Data Analysis

A. Electrical Properties During Heating

For Pt/C/HfO_2/Si devices, the capacitance has been shown to increase while the resistance decreases.[2] To demonstrate this trend, the capacitance and resistance are plotted versus temperature in **Figure S11A** and **Figure S11B**, respectively.

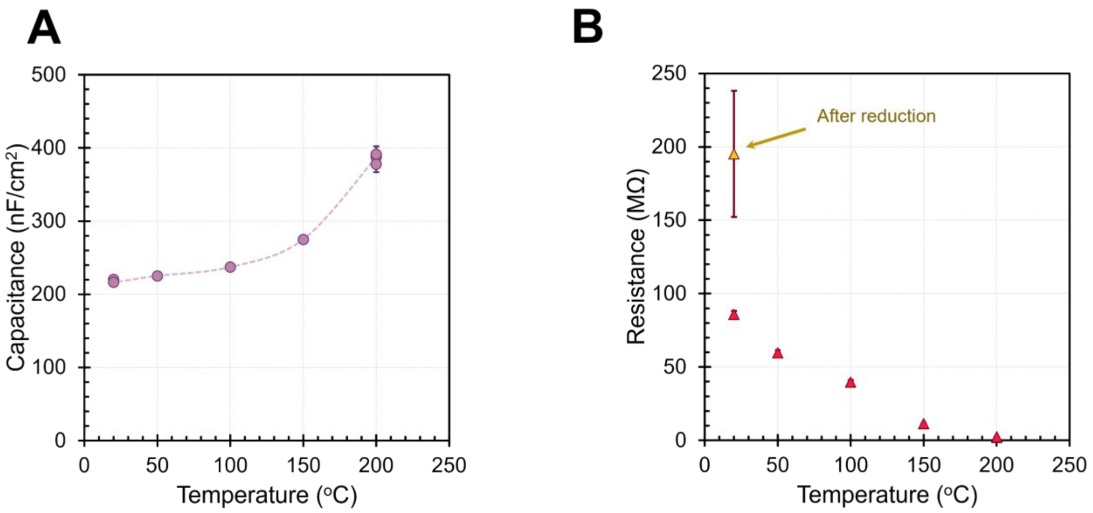

Figure S11: **(A)** The capacitance of the Pt/C/HfO_2/Si devices as the temperature increases showing that capacitance increases with temperature. **(B)** The resistance of the devices versus temperature which decreases with increasing temperature. Note that room temperature resistance of a device can be increased after a heating and reduction cycle.

Beyond the capacitance and resistance changing with temperature, the devices also experience negative current during elevated temperatures while holding 0 V. This current is often referred to as the baseline in the main text, and eliminating it is important for measuring consistent currents and integrated current peaks. To investigate the origin of these currents, we measured the current across the device while holding 0 V during the heating process. **Figure S12** below shows a typical plot of this. Note that the current peaks are negative at -8 x 10^{-8} A around the same time point that the temperature begins to approach it's setpoint (temperature is not shown). The fact that the current then begins to decline towards 0 A suggests that the driving force for this baseline is decreasing. We believe this is actually due to the equilibration of charge across the condenser which can result in a voltage across the electrodes. In turn, the HfO_2 experiences an electric field which would preferentially cause O^{2-} diffusion consistent with previous literature.[6,7] This solid-state diffusion is expected to be slow (time scale in the hours) but activated and thus should be faster

at higher temperatures. However, as the diffusion continues, its driving force should also decrease. Therefore, we believe that a significant degree of this baseline is due to ion diffusion through the dielectric, and we found that leaving the device at 0 V for a long time (overnight) or applying a negative voltage would eliminate this baseline effect.

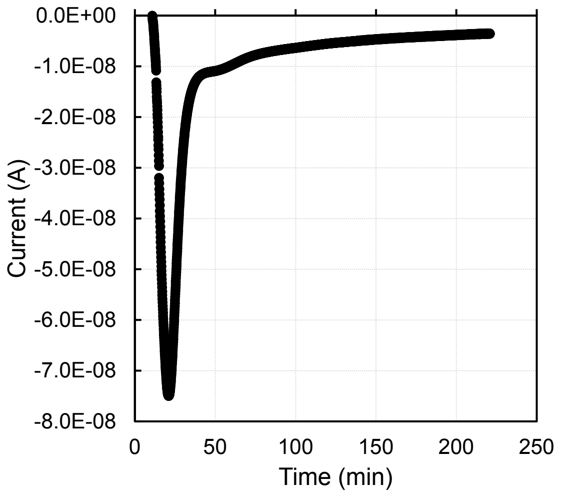

Figure S12: Experimental current versus time data while heating a Pt/C/HfO_2/Si device.

Due to the exponential nature of this baseline, the baseline never reached 0 A. For most experiments reported, it had decreased to about 10 pA which was enough to resolve the peaks of these experiments. Notably though, when the reactor would heat, a negative peak could form and when it would cool a positive peak would form. Although this could be a Seebeck effect due to changing in heating rates across the device, this would also be consisted with the diffusion mechanism which shows that increasing the temperature would increase the rate of diffusion (i.e., a more negative current) and vice versa. However, the thermal currents were 10 pA or less in all experiments with some of these effects shown in **Section S5D** below.

B. <u>IET Raw Data Analysis Methods</u>

Three devices were used for the IET experiments: Device #1 and #3 at the University of Minnesota and Device #2 at the University of Houston. Prior to each trial, the device was reduced in 50 sccm H_2 at the temperature during that trial, and a slow exponentially decaying positive peak occurred. This reduction would lead to a large positive peak caused a slowly decaying baseline. Prior to starting the IET experiment, this baseline was allowed to decay. Although this baseline was then small in comparison to the peaks the baseline still needed to be corrected to properly integrate the current. The baselines were drawn as a line to

not overfit any effect but were drawn such that current at the end of the cycle declined into the baseline since it is assumed that the current transferred should have reached steady state by the end of these trials. We speculate that the difference in baselines is related to the shift in surface coverages. The electrochemical potential of the Pt is different in highly H-covered surfaces as compared to more vacant surfaces. Since the overall baseline for these trials was minimized in 1 atm H_2 environments (after the reduction), we expect the cycles in 1 atm H_2 to reach nearly 0 A. In contrast, the Pt in the 0.005 atm H_2 cycles would have a different electrochemical potential than when the device's baseline was minimized which would mean that there was a driving force for oxygen vacancies in the HfO_2 to migrate. We believe that the current does not return to 0 A in the 0.005 atm H_2 cycles because of this speculated oxygen vacancy diffusion, but more experiments must be conducted to confirm this. A representative trial of raw current data (**Figure S13A**), the baseline-adjusted current (**Figure S13B**), and the integrated charge versus time via trapezoid rule (**Figure S13C**).

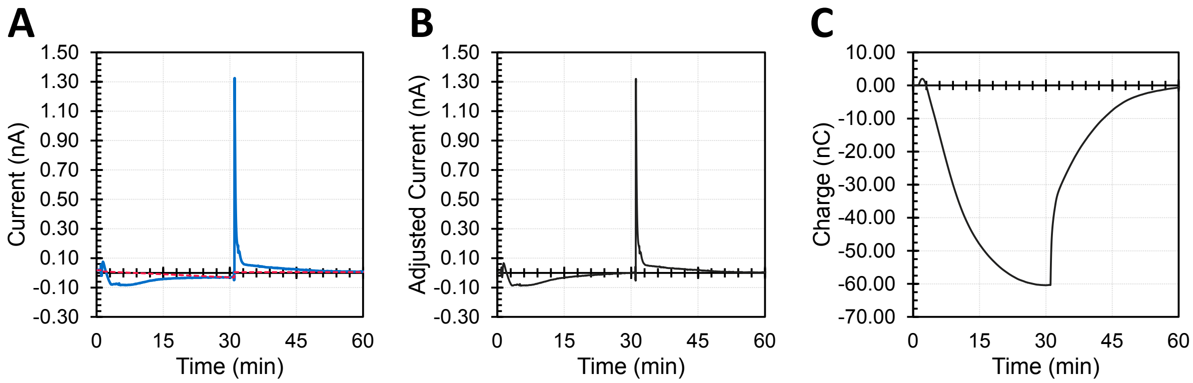

Figure S13: **(A)** The raw current data for one cycle of Device #3 at 200 ^oC showing the proposed baseline as a red dashed line. The inlet gas was switched from 1 atm H_2 to 0.005 atm H_2 at t = 1 min and then switched back to 1 atm H_2 at t = 31 min **(B)** The current data adjusted to the baseline versus time. **(C)** The integrated charge versus time resulting from the adjusted current.

C. *IET Charge Integration Data Tables*

After adjusting for these baselines, the integrations were completed using the SquidStats built in software and by subtracting out the trapezoidal area of the baseline. The integrations between the end and beginning of the 30-minute cycles (e.g., integration for the desorption peak in **Figure S13C** would be about -60 nC) are reported below in **Table S4** for Device #1 at the University of Minnesota, **Table S5** for Device #2 at the University of Houston, and **Table S6** for Device #3 at the University of Minnesota. The values in these figures along with the calculated 95% confidence intervals on these numbers are shown in **Figure 3D** in the main text.

Table S4: The integrations of the adsorption peaks for Device #1 at University of Minnesota which followed the adsorption and reset procedure. The values in the middle of the table all have the units of nC.

Cycle	200 °C	175 °C	150 °C	125 °C	100 °C
#1	61.0	41.5	22.4	17.3	5.9
#2	57.7	42.3	22.5	18.0	5.4
#3	56.5	38.5	26.6	15.9	5.3
#4	58.6	39.3	25.4	15.5	6.0
#5	59.2	41.2	26.3	--	5.7
#6	64.3	--	--	--	--
Average	59.1	40.3	24.6	16.7	5.6
95% CI	2.93	2.76	2.54	1.89	0.4

Table S5: Integrated peaks and average / 95% CI for Device #2 at the University of Houston. Values in the middle of the table are in units of nC.

Cycle	200 °C	175 °C	150 °C	125 °C	100 °C
#1	65.3	44.4	36.8	8.7	4.7
#2	74.6	54.6	26.7	9.1	3.8
#3	66.2	46.8	31.9	17.1	2.7
Average	68.7	48.6	31.8	11.6	3.7
95% CI	12.8	13.3	12.5	11.8	2.4

Table S6: The integrations of the adsorption peaks for the adsorption and desorption IET experiments for Device #3. The "Peak" column indicates the peak number in each temperature trial. The values in the middle of the table all have the units of nC. Note: the device lost conductivity prior to attempting the 100 °C trial.

Cycle	200 °C	175 °C	150 °C	125 °C
Desorption #1	-58.78	-40.13	-30.63	-20.85
Adsorption #1	61.04	41.66	31.93	21.03
Desorption #2	-63.93	-40.69	-30.38	-22.14
Adsorption #2	63.56	41.01	30.71	22.15
Desorption #3	-60.26	-41.40	-29.47	-22.36
Adsorption #3	59.60	40.64	30.70	22.08
Desorption #4	-60.37	--	--	--
Adsorption #4	59.89	--	--	--
Desorption #5	-57.89	--	--	--
Adsorption #5	56.62	--	--	--
Desorption #6	-56.17	--	--	--
Adsorption #6	57.53	--	--	--
Avg. Desorption	-60	-41	-30	-21.8
95% CI	3	2	2	2
Avg. Adsorption	60	41	31	21.8
95% CI	3	1	2	2
Avg. Combined	60	41	31	21.8
95% CI	2	1	1	0.7

D. *Titration Control Experiments*

A control experiment for the IET method with a device that can no longer store charge is shown below in **Figure S14A**. The data shown are for an IET adsorption and reset experiment at 200 °C where the current spikes upon introducing H_2 were normally in the 10^{-8} A range. In contrast, no spike was observed when switching to H_2 (at t = 5230 s) in this experiment. The currents remained at 10^{-12} A range which has been consistent with devices that can no longer store charge effectively. This is further demonstrated in **Figure S14B** which shows a CV measurement at 1.00 V/s at 200 °C. The currents are 4 orders of magnitude lower than they normally are at this temperature, and the CV curve is no longer "boxy" indicating that charge is leaking through the film at a similar rate to which it can be supplied. This device failure mode is consistent with the Pt/C layer becoming more resistive which is discussed further in **Section S5E**.

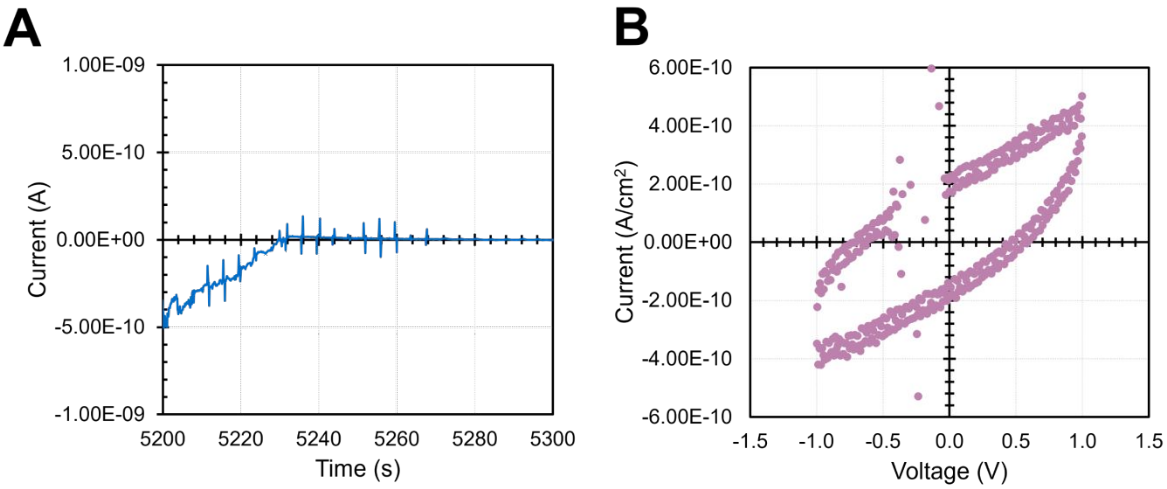

Figure S14: **(A)** A plot of currents vs. time for a IET adsorption and reset experiment at 200 ^{o}C when a device is no longer capable of storing charge. H_2 was dosed into the system at about t = 5230 s. The small, nearly instantaneous spikes in the current are likely due to oversampling since the sampling frequency was every 0.1 s in this period. **(B)** A plot of current versus voltage during a 1.00 V/s CV experiment directly after this IET trial showing that the currents are low (10^{-10} A when a normal CV on these devices show currents of 10^{-6} A at these temperatures) and that there is little hysteresis between the forward and reverse sweeps.

A control experiment was conducted to determine if the pressure change associated with switching gas streams influenced the current measured during IET experiments. Two streams of 100% H_2 were flown over the condenser at 50 sccm at 473 K such that a change between gas streams would not result in a net change in adsorption or desorption. The results, as shown in **Figure S15**, indicate that significantly less charge was condensed from actuating between two identical streams of 100% H_2 in comparison to switching between 100% and 0.5% H_2 streams. Thus, the quantified charge transfer events cannot be explained by a hydrodynamic effect.

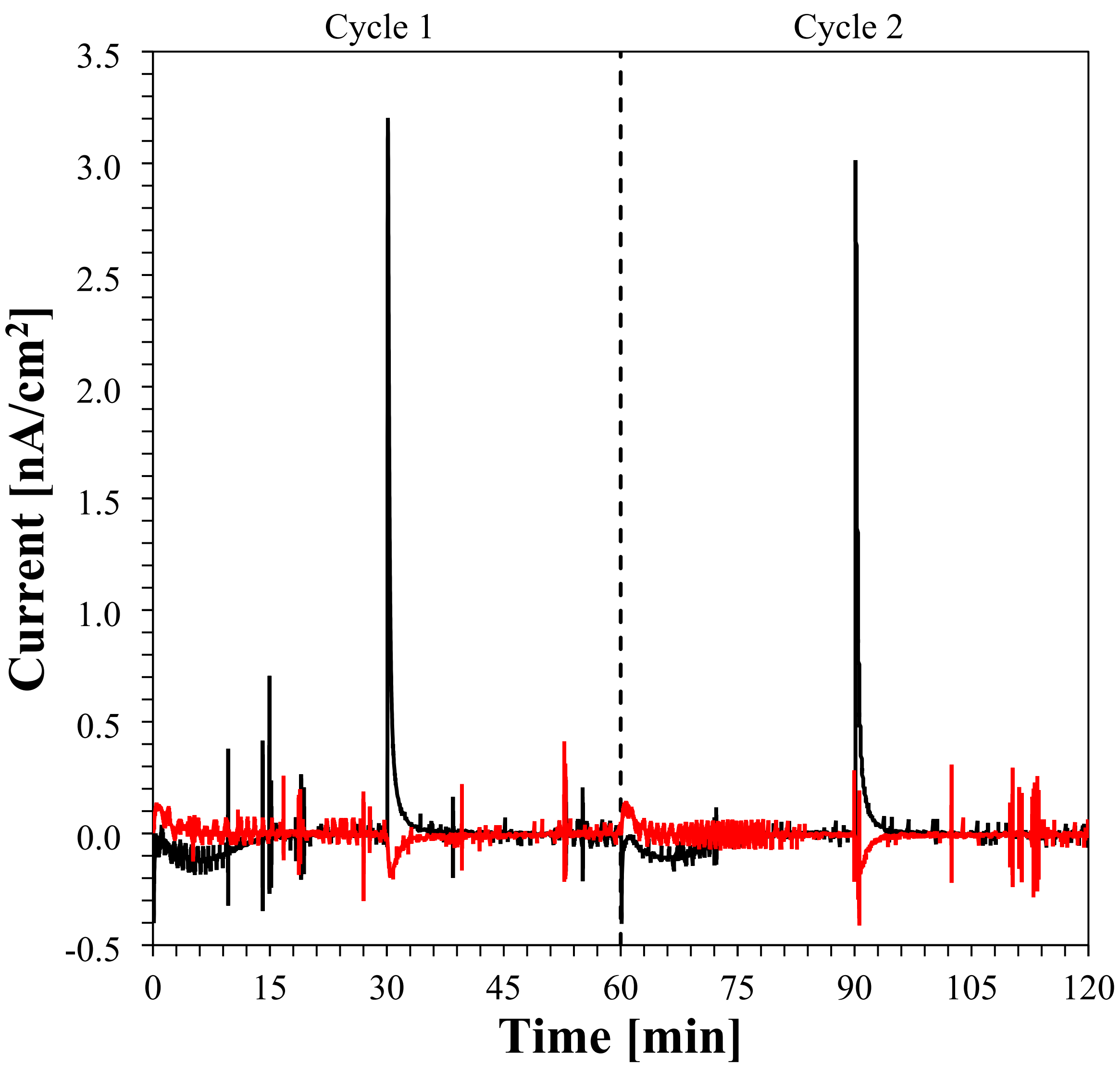

Figure S15: Chronoamperometry of Pt catalytic condenser Device #2 in 1 atm H_2 / H_2 (50 sccm) at 473 K (red) or 1 atm 100% H_2 / 0.5% H_2 (50 sccm) at 473 K (black). Time zero and every 30 minutes indicate when the gas was switched back and forth from gas stream A / gas stream B.

Another control experiment was completed to rule out thermal conductivity effects. The difference in thermal conductivity between the probe gas and balance gas, H_2 and N_2, is 231.9 mW m^{-1} K^{-1} (**Table S7)**. To ensure the charge transfer quantified was not reflective of differences in thermal conductivity between the two gases, a control experiment was conducted by switching between two inert gases with

thermal conductivities reflective of H_2 and N_2. He was selected based on its similar thermal conductivity to H_2.[8]

Table S7: Thermal conductivities of H_2, He, and N_2.

Gas	Thermal Conductivity at 500 K [mW m^{-1} K^{-1}][8]
H_2	270.9
He	221.4
N_2	39.0

The results, as shown in **Figure S16**, indicated that while peak formation occurred, it was opposite in direction compared to experimental data (100% H_2 / 0.5% H_2). To understand whether the peak formation in the control experiment can be attributed to a change in thermal conductivity of the gases, the same control experiment was conducted using two streams of 100% N_2 that came from the same ultra-high purity (UHP) N_2 tank, as shown in **Figure S17**. The results showed that peak formation also occurred when two gaseous streams with the same thermal conductivity entered the reactor. The same effect was also observed during a preliminary trial when mass flow controllers were used in place of the switching valve for a sudden controlled interruption of flow of hydrogen and inert between rich and lean activities of hydrogen in the reactor. Due to the generated change in pressure from changing flowrates, it was thus posited that an electrostatic effect or mechanical stress could have affected the measurement. The same control was run at 448 K and 298 K, where it was found that no discernable change in current outside of noise was measured, as shown in **Figure S18**. This suggests the effect was only present in the case of accelerated temperatures, and the contribution of the posited effect was at most 10% of the measured charge. Therefore, the measured charge transfer in 100% H_2 / 0.5% H_2 experiments was not significantly influenced by the difference in thermal conductivities of the gases, and while an electrostatic or mechanical effect may have been present at 473 K, it did not generate a significant enough current to change the measured extent of charge transfer value.

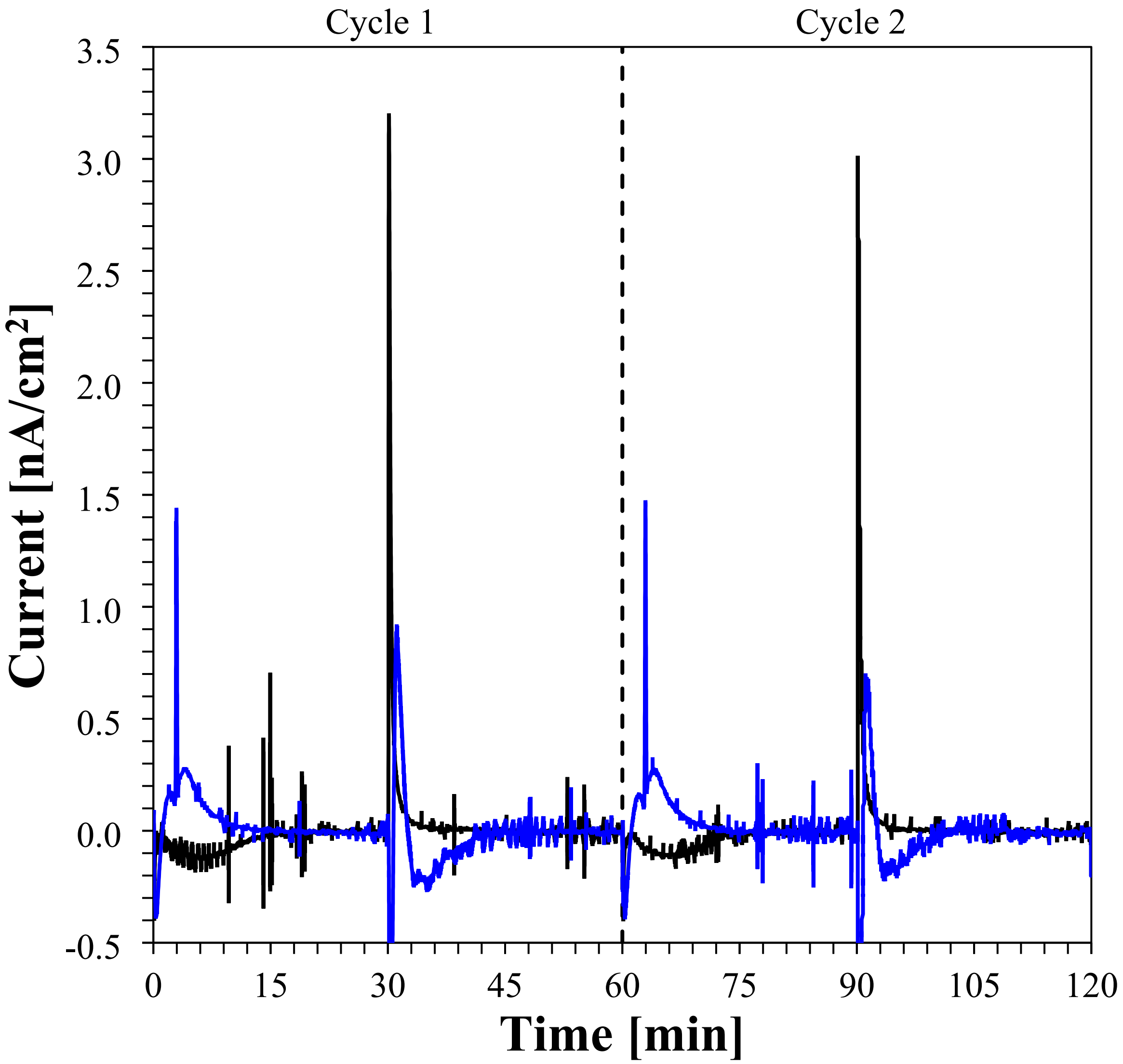

Figure S16: Chronoamperometry of Pt catalytic condenser Device #2 in 1 atm He / N_2 (50 sccm) at 473 K (blue) or 1 atm 100% H_2 / 0.5% H_2 (50 sccm) at 473 K (black). Time zero and every 30 minutes indicate when the gas was switched back and forth from gas stream A / gas stream B.

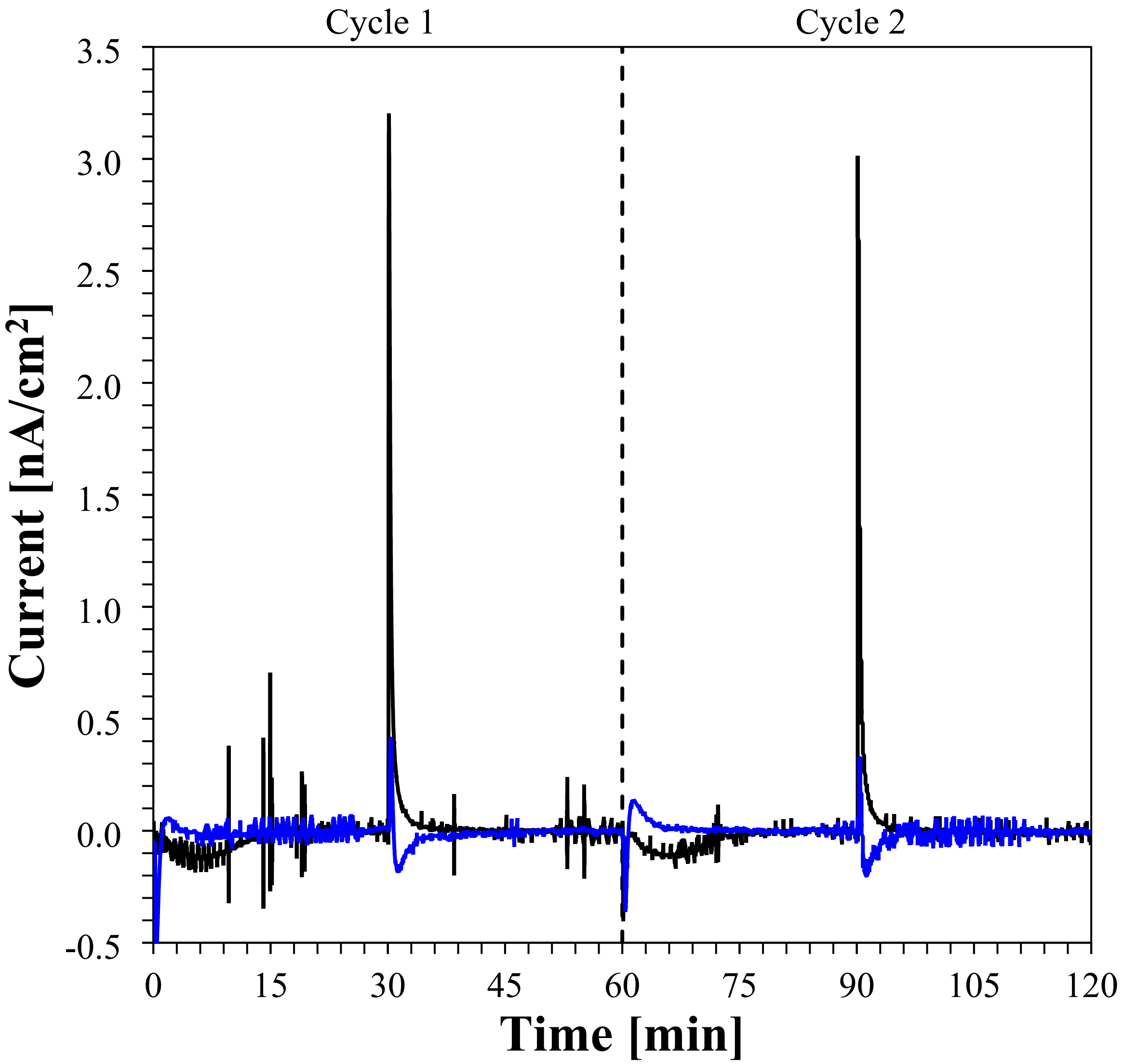

Figure S17: Chronoamperometry of Pt catalytic condenser Device #2 in 1 atm N_2 / N_2 (50 sccm) at 473 K (blue) or 1 atm 100% H_2 / 0.5% H_2 (50 sccm) at 473 K (black). Time zero and every 30 minutes indicate when the gas was switched back and forth from gas stream A / gas stream B.

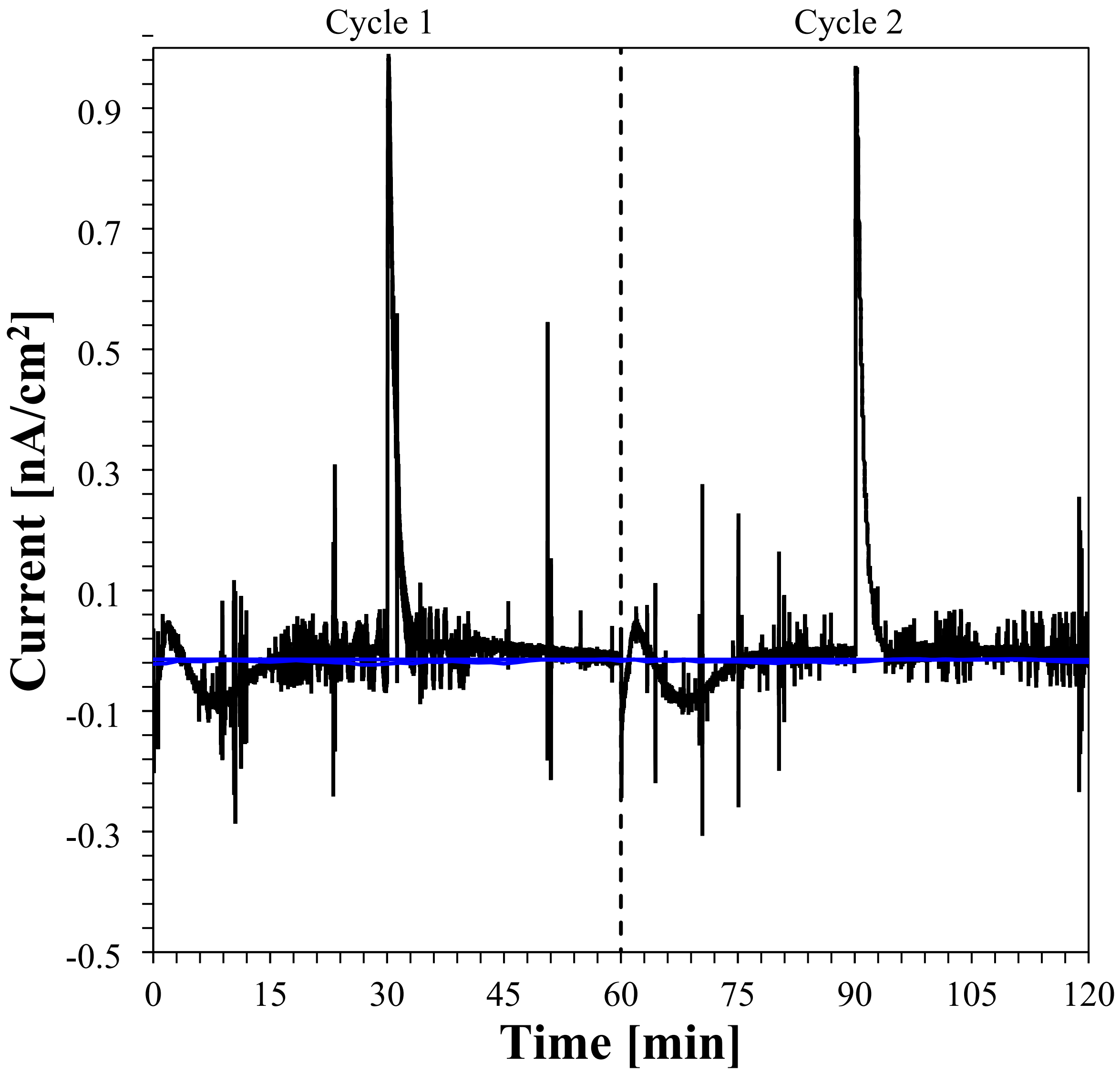

Figure S18: Chronoamperometry of Pt catalytic condenser Device #2 in 1 atm N_2 / N_2 (50 sccm) at 448 K (blue) or 1 atm 100% H_2 / 0.5% H_2 (50 sccm) at 448 K (black). Time zero and every 30 minutes indicate when the gas was switched back and forth from gas stream A / gas stream B.

E. Device Stability and Failure Modes

As outlined by the case above, the electronic integrity of the condensers are paramount to making repeatable IET measurements. Because of this CVs were taken frequently to monitor for significant changes in the devices ability to store charge. As mentioned previously, as long as there is significant hysteresis

between the forward and reverse sweeps (i.e., the currents are equal and opposite in value upon the forward and reverse sweeps) and the overall resistivity wasn't too high like the case in **Figure S14**, then the IET measurements were repeatable. To this end, representative CVs were chosen for the devices at the temperatures shown throughout the study and are reported in **Figure S19**. For these devices, the capacitance would slowly decrease over time spent in the reactor (e.g., from 400 nF at 200 °C initially to 300 nF within 1 day), but the overall shape of the current vs. voltage CV curve remained consistent.

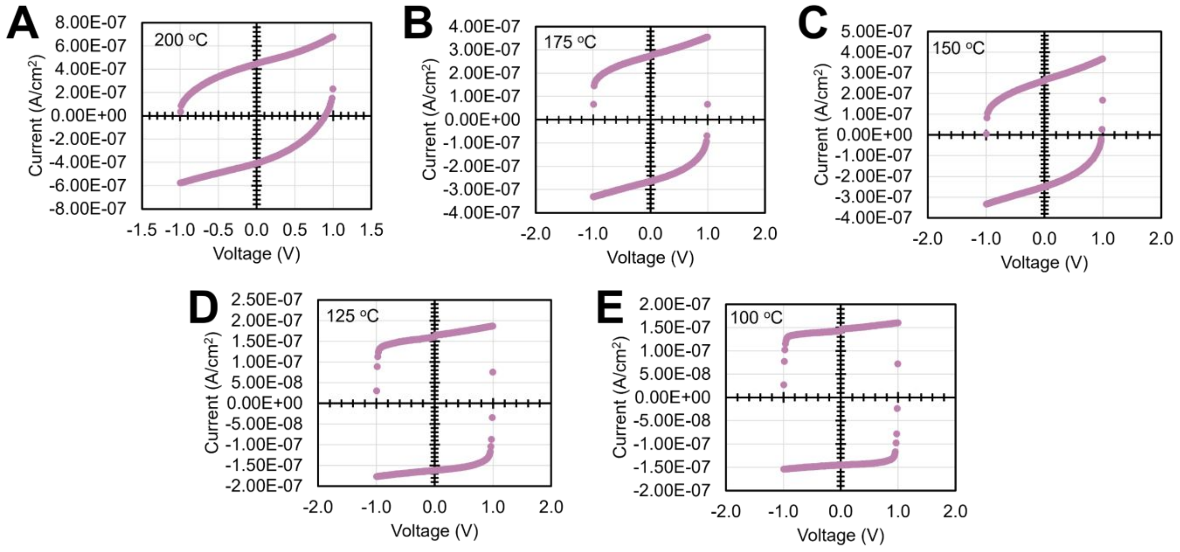

Figure S19: Typical current vs. voltage graph of 1.00 V/s CV measurements at **(A)** 200 °C, **(B)** 175 °C, **(C)** 150 °C, **(D)** 125 °C, **(E)** 100 °C.

In general, there were two failure modes to monitor for the device's electronic properties. Firstly, if excessive voltages or currents are applied to the device or the device gets scratched, the HfO_2 film can breakdown resulting in low resistances (high currents). Interestingly, this failure mode could be healed by heating and reducing the device as long as the short wasn't severe (i.e., mA currents at 1 V). The second failure mode involved the Pt/C film increasing in resistance such that it reached resistances on the same order of magnitude as the HfO_2. This is the way that the device failed in **Figure S14**, and seems to be permanent. The resistance of the Pt/C can easily be tested using sheet conductance measurements by placing both the working and counter electrodes on the Pt/C. When the Pt/C loses conductivity the currents returned by sheet conductance are much lower. We are uncertain of why this failure mode occurs but it could be that the Pt or C agglomerate gradually over time. It is unclear whether specific gas environments (N_2 vs. H_2) affect this process, but lower temperatures seem to slow this mode of failure.

S6. Hydrogen Adsorption

A. Hydrogen Coverage

To convert between the temperature x-axis in **Figure 4D** and the x-axis of H atom adsorbed in **Figure 4E**, literature values for adsorption free energies were assumed. In all cases, the reference pressures (P°) used during the calculations was 1 atm. Hydrogen coverages were calculated based on equilibrium expressions relating the partial pressure of H_2 (P_{H_2}) and the coverage of H* (θ_H) to an adsorption enthalpy (ΔH) and entropy (ΔS), as shown by **Equations 4** and **5** in the main text. To arrive at the final values of "Additional H Atom Adsorbed", hydrogen coverage was converted to absolute quantities by assuming a device area of 1 cm^2 (area exposed by the shadow mask during Pt and C deposition) and 10^{15} adsorption sites/cm^2.

However, there may be surface roughness that increases the total surface area past 10^{15} cm^{-2}. In the absence of measuring the surface roughness to the nanometer scale through techniques like AFM, we are left to speculate about how much surface area there is. From our previous reports, TEM images showed some surface roughness but not likely enough to increase the order of magnitude of sites past 10^{15}. Additionally, a rough surface is likely to expose lower packing crystal facets (i.e., they have site densities less than 10^{15} cm^{-2}) which means that the decreased average site density combined with the increased surface area may even out to around 10^{15} cm^{-2}. Nonetheless, future work should directly measure the exposed number of sites to take the final calculation of amount of H adsorbed from an estimate to a more exact number.

B. Main Reference for Adsorption Values

The primary reference used to identify ΔH^o and ΔS^o for use in **Equation 4** in the main text was from García-Diéguez, Hibbitts, and Iglesia (2019).[9] We chose to primarily use this literature source because (1) it had a Pt particle size similar to the expected particle size of our films (1-2 nm) and (2) it had the most extensive range of experimental data we could find. Using the data reported therein for 1% Pt/SiO_2, the values were calculated to be $\Delta H^o = -64$ kJ/mol and $\Delta S^o = -70.8$ J/mol K. Using these values in **Equation 5** in the main text, we then calculated the coverage at various partial pressures of H_2 as shown in **Figure S2A**. Subtracting from coverages at various partial pressures of H_2 from the coverage at 1 atm H_2 returned the values shown in **Figure S20B** showing that there is a larger change in adsorption at higher temperatures until the coverages at 1 atm H_2 begin to decline (around 300 ^{o}C).

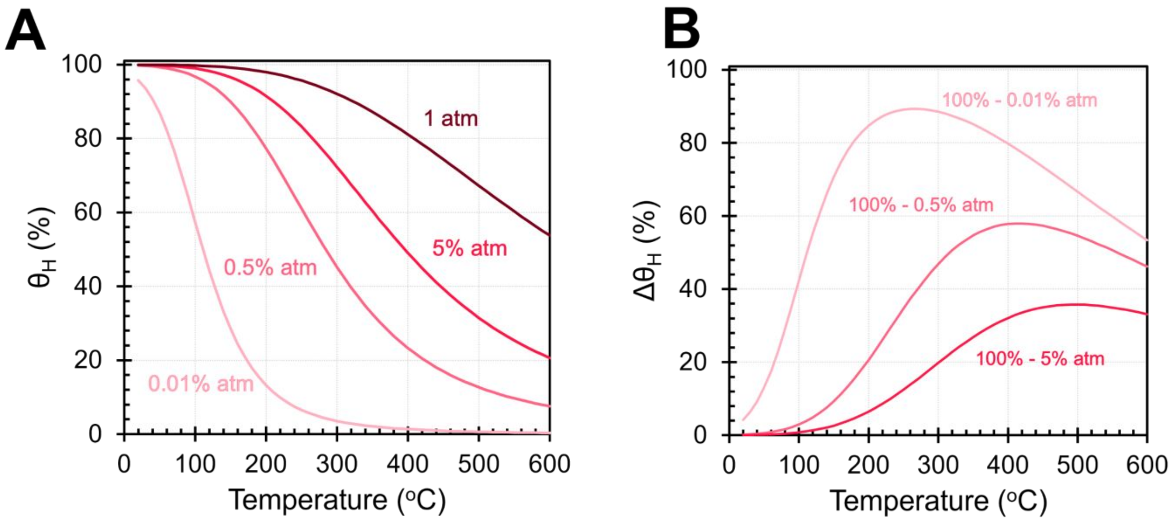

Figure S20: **(A)** Calculated hydrogen coverage as a function of temperature for 100%, 5%, 0.5%, and 0.01% atm partial pressures H_2 using ΔH^o = -64 kJ/mol and ΔS^o = -70.8 J/mol K. **(B)** The difference in coverage between 100% atm H_2 and the other three partial pressures from subpanel **S20A** vs. temperature.

From this coverage data, the amount of H* adsorbed was calculated using **Equation S5** below where

$$n_H = \frac{10^{15}\ \text{sites}}{\text{cm}^2} * \frac{1\ \text{pmol}}{6.02*10^{23}*10^{-12}} * 1\ \text{cm}^2 * \left(\theta_H(1\ \text{atm,T}) - \theta_H(0.005\ \text{atm,T})\right) \quad \text{(S5)}$$

Using this number of additional H atoms adsorbed, the partial charge transfer number can be estimates as the slope of the electrons transferred versus the change in adsorbates shown in **Equation S6**. Where F is Faraday's constant

$$\int_0^t I\ dt = \delta F n_H \quad \text{(S6)}$$

C. *Work Function Change of Pt upon H2 Adsorption*

The work function (the amount of energy required to pull an electron from inside a material to infinitely far away from the defined surface) changes linearly with the extent of charge transfer of adsorbates on a surface (Eq. S7). Looking at single-crystal literature on change of Pt work function upon H_2 adsorption, it is noted that there is a linear trend across the coverage range of this work (75% to 100%, **Figure S21**). It is therefore expected that a single value of charge transfer would be measured across the coverage range of this work.[10–12] Furthermore, through taking the difference in work function changes over the coverage range of this work (0.75-1.0), one can predict the work function change. A change in work function can be predicted from this work using Equation S7, where $\Delta\Phi$ is the change in work function, δ_{i0} is the extent of charge transfer near zero coverage, d is the surface adsorbate bond length, Γ_s is the sites per unit area (10^{19} sites/m^2), θ_i is the coverage, α is the polarizability of the bond, e is the fundamental charge (1.602×10^{-19} C), and ε_0 is the vacuum permittivity (8.85×10^{-12} F/m). The best fit to the change in work function data from Christmann, Ertl, and Pignet[11] (using a bond length of 2 Å) resulted in an δ_{i0} = 0.27% |e|/H and α = -0.036 eÅ^2/V. The α value is non-physical, which the authors of that report acknowledged. DFT-based values (δ_{i0} = 0.6% |e|/H and α = 0.003 eÅ^2/V) were also plotted in **Figure S21** to show that the experimental work function data matched theory.[12]

$$\Delta\Phi = \frac{(\delta_{i0}, ed)e\Gamma_s\Delta\theta_i}{\varepsilon_0(1 + \frac{\alpha\Gamma_s}{d\varepsilon_0}\theta_i)} \quad \text{(S7)}$$

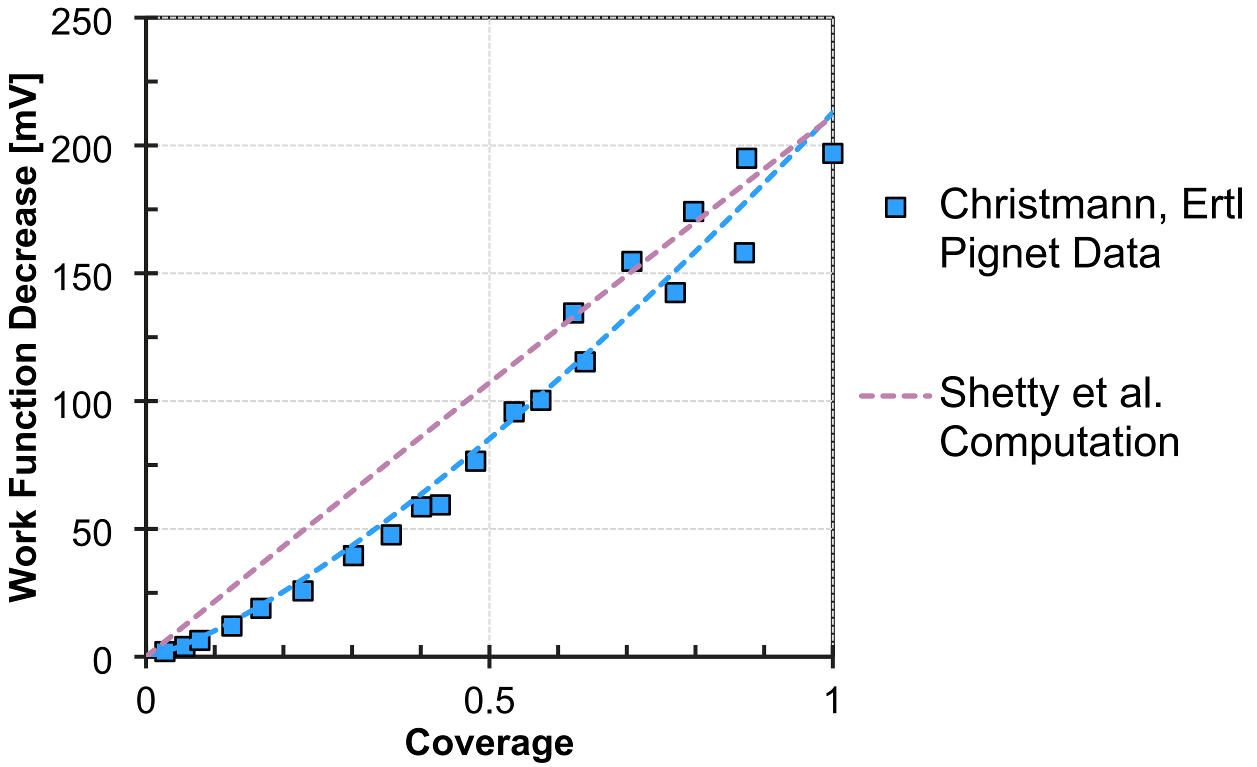

Figure S21: Comparison of effect of hydrogen adsorption on work function from Christmann, Ertl, and Pignet[11] (■) supplemented by computational data from Shetty et al.[12] (-).

D. Sensitivity to ΔH *and* ΔS

Since the calculated coverage values were sensitive to enthalpy and entropies of adsorption values assumed, additional literature was considered to determine a range of possible thermodynamic adsorption values. **Table S8** below describes a list of experimental adsorption values not including the main reference.

Table S8: Collection of platinum particle sizes, loading percentages, supports, and thermodynamic values.[9,13–15] Unlisted entropies of adsorption were estimated based on a compensation effect (see **Figure S22**).

Loading/Support	d_p^* (nm)	ΔH_{ads}^o (kJ/mol)	ΔS_{ads}^o (J/mol.K)	Reference
MFI type silicalite-1	1.0	-72	-125	13
nanoparticle	1.6	-47	-56	9
nanoparticle	3.0	-40	-36	9
nanoparticle	9.1	-37	-29	9
1.2% Pt/Al_2O_3	1.1	-61	-	15
0.7% Pt/SiO_2	1.3	-55	-	15
0.7% Pt/SiO_2	1.4	-45	-	15
0.7% Pt/SiO_2	2.7	-33	-	15
0.6% Pt/SiO_2	2.8	-44	-	15

0.6% Pt/SiO_2	4.0	-47	-	[15]
2.7% Pt/SiO_2	6.5	-61	-	[15]
4.4% Pt/SiO_2	5.5	-70	-	[15]
5.0% Pt/SiO_2	3.9	-62	-	[15]
5.0% Pt/SiO_2	22	-44	-	[15]
1.7% Pt/SiO_2	1.9	-69	-	[15]
1.7% Pt/SiO_2	1.6	-67	-	[15]
1.8% Pt/SiO_2	2.0	-64	-	[15]
2.2% Pt/SiO_2	2.0	-71	-	[15]
Pt Powder	1600	-57	-	[15]

*Particle diameter

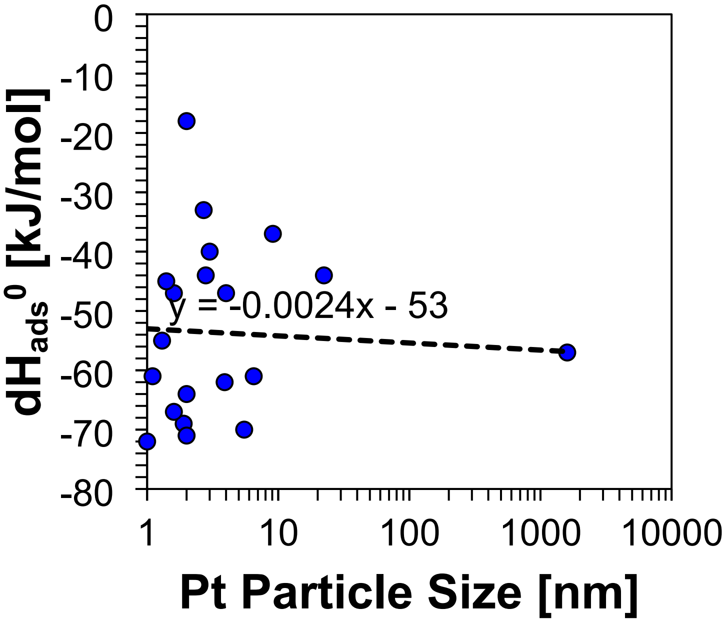

Figure S22: Collected enthalpy of hydrogen adsorption as a function of platinum particle size. Enthalpic values that were not discretely mentioned in literature were estimated graphically across a range of coverages.[9,13–15]

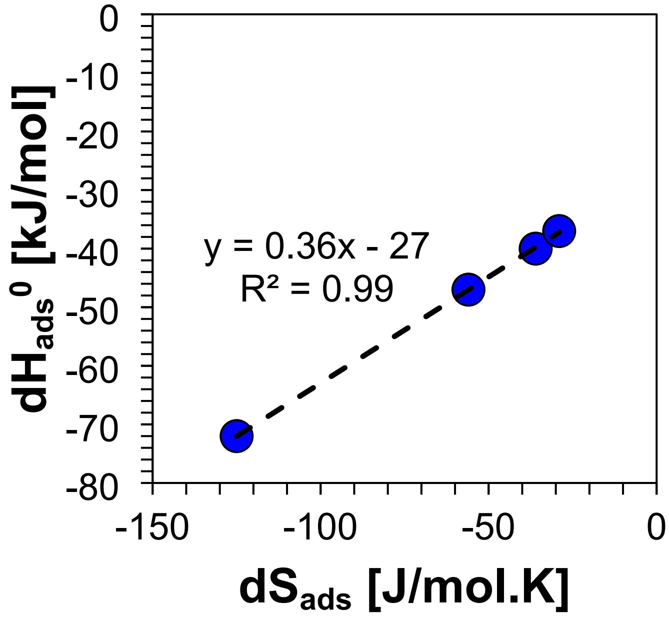

Figure S23: Enthalpy of adsorption as a function entropy of adsorption compensation effect.[9,13–15] Most enthalpies collected for hydrogen adsorption on platinum in literature fell into this range, thus the model was used to predict entropies of adsorption for literature sources that reported the enthalpy of adsorption but did not report the entropy of adsorption.

The extent of partial charge transfer between a hydrogen adsorbate and the platinum surface was estimated by comparing the cumulative electrons transferred during an adsorption/desorption event, with the quantity of adsorbates involved. As described in **Equation S6**, the total charge was calculated as the time-based integral of current during adsorption/desorption of hydrogen on the platinum catalytic condenser, which was then converted to a molar quantity of electrons using Faraday's constant.

The moles of hydrogen adsorbate resulting in the measured charge transfer were quantified by estimating the fractional coverage of dissociated hydrogen on the platinum surface at distinct hydrogen partial pressure in the gas phase, assuming a Langmuir isotherm. The heat of molecular hydrogen adsorption was taken to be the average of multiple independent literature measurements (-49 kJ/mol, **Table S8**).

Entropies of hydrogen adsorption are less readily available, which were correlated to the average heat of adsorption per a reported compensation effect (**Figure S23**), resulting in an assumed -62 J/mol.K entropy of molecular hydrogen adsorption. For the platinum surface, a site density of 10^{15} sites/cm^2 was assumed. Over several orders of magnitude in platinum particle size, the enthalpy of adsorption remained consistent, indicating that under these conditions, the enthalpy of adsorption is structure insensitive.

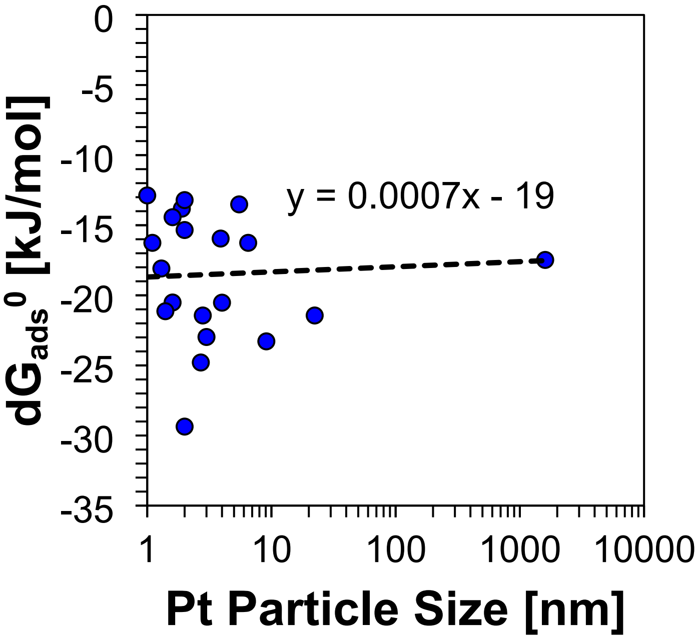

Figure S24: Free energy change of hydrogen dissociative adsorption as a function of platinum particle size.[9,13–15]

To consider the range of possible partial charge transfer number that could be calculated from these literature values, the listing with the most favorable (i.e., most negative) ΔG value, the value used reported above from literature, and the least favorable ΔG were used to calculate the pmol of additional H atoms adsorbed as was done for **Figure 4E** in the main text. Plotting the most and least thermodynamically favorable values allows us to assess the largest and smallest change in H_2 atoms adsorbed to bracket the values for the partial charge transfer number. The plots of charge transferred versus the amount of H adsorbed is shown in **Figure S25** below using Device #1's results.

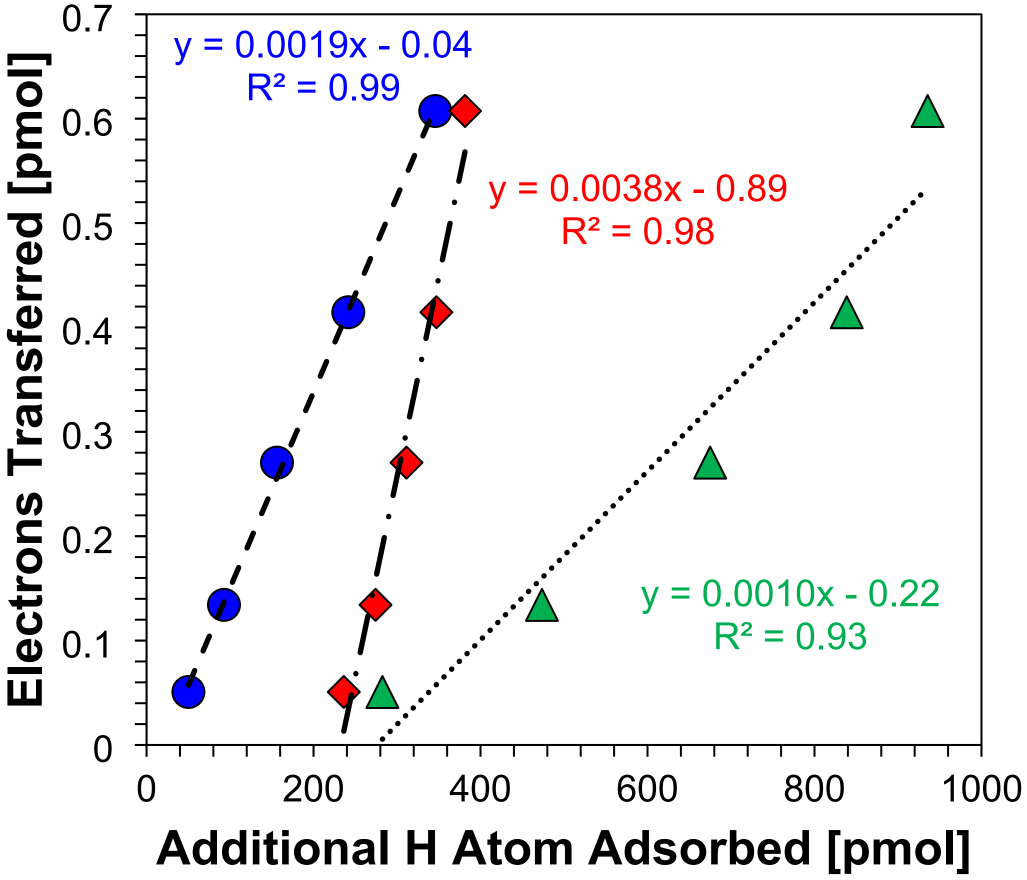

Figure S25: Partial charge transfer as a function of an additional hydrogen atom being adsorbed onto a platinum surface for the values from literature used in the main text (blue), the least favorable thermodynamics of adsorption (red), and the most favorable thermodynamics of adsorption (green). All rates of partial charge transfer per additional hydrogen atom adsorbed fall within the same order of magnitude. [9,13–15]

Overall, this analysis demonstrates that the range of values for the partial charge transfer that can be justified from our literature search is $\delta = [0.1\%, 0.4\%]$. This indicates that range of possible partial charge transfer number is quite small when considering sensitivity to reasonable ΔH and ΔS values. Notably, only the values calculated from the literature used in the main text had nearly no y-intercept on the linear fits. A

positive y-axis intercept would be unphysical under our hypothesis of the charge transfer being adsorption-based (i.e., charge transfer when there is no change in adsorption, so there must be some other charging mechanism). However, a negative y-intercept (or rather a positive x-intercept) may be possible if the polarity of the adsorbate is zero up to certain coverages.

S7. Heat Transfer Model

`Thermo-electric currents occur when two electrodes are at different temperatures, and these different temperatures cause a difference in their electrochemical potential. The result is a current driving across the electrodes. We completed a control experiment in which we switched between He and N_2 (S5.D) to demonstrate that changing the thermal conductivity of the gas alone does not generate sufficient current to explain our results. However, we developed a heat transfer model for adsorption-based surface heating to further rule out thermoelectric effects.

To do we start with the general heat equation in its 1D form, where ρ is the density, C_p is the heat capacity on a mass basis, T is the temperature, t is the time, k is the thermal conductivity (in W/m K), and x is a position unit:

$$\rho C_p \frac{\partial T}{\partial t} = k \frac{\partial^2 T}{\partial x^2} \tag{S8}$$

By assuming 1D behavior, we ignore edge effects on the device. We will further assume (1) steady state behavior, (2) that the bottom of the Si electrode (attached to the electrical stage, define as $x = 0$) is at the reactor temperature, T_R, since the stage may act as a heat sink, and (3) that there is convection and adsorption-based heat transfer occurring at all times at the top of the surface ($x = L$). By assuming that there is adsorption-based heat transfer constantly occurring, we are explicitly trying to calculate an upper bound on the surface temperature. Therefore, the simplified heat equation and the corresponding boundary conditions can be written as:

$$\frac{d^2 T}{dx^2} = 0 \tag{S9}$$

$$T(x = 0) = T_R \tag{S10}$$

$$-k\frac{dT}{dx}\Big|_{x=L} = h(T(L) - T_R) - \bar{q}_{ads} \tag{S11}$$

Here, h is the heat transfer coefficient (in W/m^2 K) and $\bar{q}_{ads}$ is the heat flux imparted to the top surface due to adsorption. Solving this equation with the listed boundary conditions yields the following linear equation for temperature within the device:

$$T(x) = T_R + \frac{\bar{q}_{ads}}{hL + k}x \tag{S12}$$

We can further expand the heat flux from adsorption by knowing including the moles of molecules adsorbing per unit area (which in turn consists of the areal site density, $\bar{N}_{sites}$, the change in coverage, $\Delta\theta$, and a factor of $\frac{1}{2}$ because the heat of adsorption is written as per H_2 adsorbed), the absolute value of the heat of adsorption (ΔH_{ads}), and the period of time it takes to adsorb the full $\Delta\theta$ (τ_{ads}). Therefore, the heat flux from adsorption and the surface temperature can be written as:

$$\bar{q}_{ads} = \frac{\bar{N}_{sites}\Delta H_{ads}\Delta\theta}{2\tau_{ads}} \tag{S13}$$

$$T(L) = T_R + \frac{\bar{N}_{sites}\Delta H_{ads}\Delta\theta}{2\tau_{ads}(hL + k)}L \tag{S14}$$

To calculate this upper bound on the surface temperature, we used the following parameters:

Table S9: Parameters, their definitions, and the values used to estimate the maximum surface temperature.

Parameter	Definition	Value	Source
$\overline{N}_{sites}$	Number of sites per unit area	1 x 10^{19} sites/m^2 =1.66 x 10^{-5} mol/m^2	See main body
$\Delta\theta$	Change in coverage	0.25	
ΔH_{ads}	Heat of adsorption	-62 kJ/mol	
k	Thermal conductivity (of silicon)	33 W/m K	Source: [16]
L	Total device thickness	0.5 mm	
h	Heat transfer coefficient	10 W/m^2 K	Lower bound, source:[17]
T_R	Reactor temperature	200 ^{o}C	
τ_{ads}	Turnover time constant for adsorption (e.g., the period over which one full round of adsorption takes)	1 ms	

Using these parameters, we can estimate the surface temperature to be 2 mK above the reactor temperature, and is likely very low because of the high conductivity of the Si layer (which is 10,000x thicker than the other components and will dominate conduction). This represents an upper bound because (1) the heat transfer coefficient was chosen to be a lower bound, and (2) adsorption would not continuously take place as we let it in this model. Additionally, the Seebeck coefficient of p-doped Si is around 200 µV/K.[16] With a temperature increase of 2 mK and a typical resistance across the catalytic condenser devices (1 MΩ), there would only be a 0.05 µV drop across the device resulting in currents of about 5 x 10^{-13} A which is well below the currents observed during IET experiments. Therefore, we believe that the current measurements were free of thermoelectric currents derived from adsorption-based surface heating.

S8. Reactor Dynamics Model to Explain Current Response

To understand the adsorption and desorption profiles, the current response was related to coverage changes with respect to time ($\frac{d\theta}{dt}$), which in turn is correlated with hydrogen partial pressure, that can be described using chain rule. $\frac{dP_{H_2}}{dt}$ represents the change in hydrogen partial pressure with respect to time, and $\frac{d\theta}{dP_{H_2}}$ represents the change in coverage with respect to hydrogen partial pressure.

$$I \propto \frac{d\theta}{dt} = \frac{dP_{H_2}}{dt} * \frac{d\theta}{dP_{H_2}} \quad \text{(S15)}$$

The current profile was dictated by both reactor hydrodynamics (i.e., accumulation of partial pressure) and H_2 adsorption thermodynamics. The integrated current with time would still be solely dictated by the overall change in coverage. To model this for the University of Minnesota reactor, we assumed the volume to behave like a continuously stirred tank, consistent with residence time distribution (RTD) measurements (**Section S2D**). The total volume was set to 160 mL and a gas flow rate of 50 sccm, with inlet hydrogen partial pressures of 0.005 atm H_2 at t = 0 min and 1 atm H_2 at t = 30 min. The temperature was set to 200 ^{o}C, and the adsorption thermodynamics from García-Diéguez, Hibbitts, and Iglesia were used.[9] The hydrogen partial pressure in the volume (P(t)) was therefore calculated as,

$$P(t) = P_{inlet} + [P(t{=}0) - P_{inlet}]e^{-\frac{Qt}{V}} \quad \text{(S16)}$$

Figure S26A shows the modeled partial pressures and resulting H* coverage as functions of time. The partial pressure follows exponential decays to the new partial pressures value with time constant τ = Volume/flowrate of about 2 min. **Figure S26B** below shows the derivative of coverage with respect to time versus time which we expect to be a good indicator of the current shape. **Figure S26C** shows the adjusted current shown in **Figure S13**. There is indeed good agreement between the experimental and modeled current behavior. Considering the coverage versus time plot, adsorption currents are sharper given that the hydrogen partial pressure required to saturate the surface are almost immediately reached when switching to 1 atm H_2. In contrast, the coverage decreases gradually when the reactor is being depleted of hydrogen because more time is spent transitioning through partial pressures where the coverage is greatly changing.

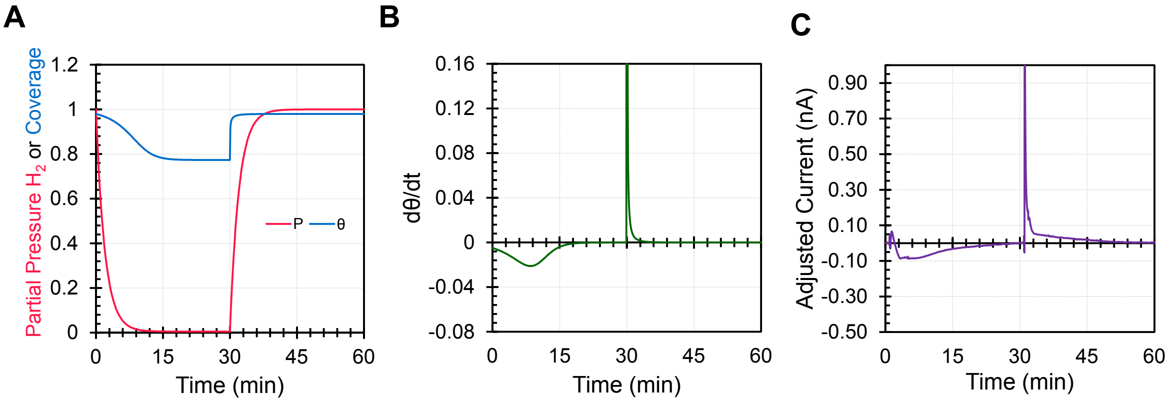

Figure S26: **(A)** The modeled partial pressure (red) and H* coverage (blue) versus time using T = 200 ^{o}C, V = 160 mL, Q = 50 sccm, P_{high} = 1 atm, P_{low} = 0.005 atm, and thermodynamics covered in **Section S6B**. **(B)** The change in H* coverage with respect to time, dθ/dt, according to the modeled coverage. **(C)** The experimental adjusted current from **Figure S13**, zoomed in to show the desorption peak better.

S9. Pt and Si Charge Equilibration Analysis

An important assumption is that the charge transfer measured is equal to the partial charge transfer between H* and Pt (δ); all electrons transferred from H* to Pt are measured as current flow. To claim that $\delta \approx \int I\, dt$, the amount of charge Pt transferred to the p^{++}-Si must not be enough to sufficiently change the charge carrier density of the Si. In the catalytic condensers, the thickness of Pt is approximately 1 nm while the thickness of the p^{++}-Si is reported as 0.5 mm. Considering the reported resistivity of the p^{++}-Si is no less than 0.005 Ω cm, 10^{19} charge carriers/cm^3 were estimated as a minimum.[18] Combining this with the thickness, 5 x 10^{17} charge carriers are estimated per cm^2 of device area. Considering that the maximum charge transferred during any one IET measurement was 7.8 x 10^{11} e^-/cm^2 (125 nC), the charge transferred is six orders of magnitude smaller than the total amount of carriers in p^{++}-Si. Therefore, the electronic properties of p^{++}-Si are not expected to be affected by the charge transfer and can be treated as a sink.

References

(1) Onn, T. M.; Gathmann, S. R.; Wang, Y.; Patel, R.; Guo, S.; Chen, H.; Soeherman, J. K.; Christopher, P.; Rojas, G.; Mkhoyan, K. A.; Neurock, M.; Abdelrahman, O. A.; Frisbie, C. D.; Dauenhauer, P. J. Alumina Graphene Catalytic Condenser for Programmable Solid Acids. *JACS Au* **2022**, *2* (5), 1123–1133. https://doi.org/10.1021/jacsau.2c00114.

(2) Onn, T. M.; Gathmann, S. R.; Guo, S.; Solanki, S. P. S.; Walton, A.; Page, B. J.; Rojas, G.; Neurock, M.; Grabow, L. C.; Mkhoyan, K. A.; Abdelrahman, O. A.; Frisbie, C. D.; Dauenhauer, P. J. Platinum Graphene Catalytic Condenser for Millisecond Programmable Metal Surfaces. *J. Am. Chem. Soc.* **2022**, *144* (48), 22113–22127. https://doi.org/10.1021/jacs.2c09481.

(3) Oh, K.-R.; Onn, T. M.; Walton, A.; Odlyzko, M. L.; Frisbie, C. D.; Dauenhauer, P. *Fabrication of Large Area Metal-on-Carbon Catalytic Condensers for Programmable Catalysis*; preprint; Chemistry, 2023. https://doi.org/10.26434/chemrxiv-2023-bt10w.

(4) Onn, T. M.; Oh, K.-R.; Adrahtas, D. Z.; Soeherman, J. K.; Hopkins, J. A.; Frisbie, C. D.; Dauenhauer, P. J. Flexible and Extensive Platinum Ion Gel Condensers for Programmable Catalysis. *ACS Nano* **2023**, acsnano.3c09815. https://doi.org/10.1021/acsnano.3c09815.

(5) Fogler, H. S. Chapter 13: Distributions of Residence Times for Chemical Reactors. In *Elements of Chemical Reaction Engineering*; Prentice Hall, 1992; pp 708–722.

(6) Kim, S.; Lee, D.; Park, J.; Jung, S.; Lee, W.; Shin, J.; Woo, J.; Choi, G.; Hwang, H. Defect Engineering: Reduction Effect of Hydrogen Atom Impurities in HfO 2-Based Resistive-Switching Memory Devices. *Nanotechnology* **2012**, *23* (32). https://doi.org/10.1088/0957-4484/23/32/325702.

(7) Sharath, S. U.; Kurian, J.; Komissinskiy, P.; Hildebrandt, E.; Bertaud, T.; Walczyk, C.; Calka, P.; Schroeder, T.; Alff, L. Thickness Independent Reduced Forming Voltage in Oxygen Engineered HfO 2 Based Resistive Switching Memories. *Appl. Phys. Lett.* **2014**, *105* (7). https://doi.org/10.1063/1.4893605.

(8) Huber, M.; Harvey, A. In *Thermal Conductivity of Gases*; CRC-Press, Boca Raton, FL, 2011; Vol. 92, pp 240–241.

(9) García-Diéguez, M.; Hibbitts, D. D.; Iglesia, E. Hydrogen Chemisorption Isotherms on Platinum Particles at Catalytic Temperatures: Langmuir and Two-Dimensional Gas Models Revisited. *J. Phys. Chem. C* **2019**, *123* (13), 8447–8462. https://doi.org/10.1021/acs.jpcc.8b10877.

(10) Higuchi, I.; Ree, T.; Eyring, H. Adsorption Kinetics I. The System of Alkali Atoms on Tungsten. *J. Am. Chem. Soc.* **1955**, *77* (19), 4969–4975.

(11) Christmann, K.; Ertl, G.; Pignet, T. Adsorption of Hydrogen on a Pt(111) Surface. *Surf. Sci.* **1976**, *54* (2), 365–392. https://doi.org/10.1016/0039-6028(76)90232-6.

(12) Shetty, M.; Ardagh, M. A.; Pang, Y.; Abdelrahman, O. A.; Dauenhauer, P. J. Electric-Field-Assisted Modulation of Surface Thermochemistry. *ACS Catal.* **2020**, *10* (21), 12867–12880. https://doi.org/10.1021/acscatal.0c02124.

(13) Yang, G.; Akhade, S. A.; Chen, X.; Liu, Y.; Lee, M.; Glezakou, V.; Rousseau, R.; Lercher, J. A. The Nature of Hydrogen Adsorption on Platinum in the Aqueous Phase. *Angew. Chem. Int. Ed.* **2019**, *58* (11), 3527–3532. https://doi.org/10.1002/anie.201813958.

(14) Li, Y.; Yang, R. T. Hydrogen Storage on Platinum Nanoparticles Doped on Superactivated Carbon. *J. Phys. Chem. C* **2007**, *111* (29), 11086–11094. https://doi.org/10.1021/jp072867q.

(15) Sen, B. The Influence of Platinum Crystallite Size on H2 and CO Heats of Adsorption and CO Hydrogenation. *J. Catal.* **1991**, *130* (1), 9–20. https://doi.org/10.1016/0021-9517(91)90087-K.

(16) Zhou, H.; Kropelnicki, P.; Tsai, J. M.; Lee, C. Study of the Thermoelectric Properties of Heavily Doped Poly-Si in High Temperature. *Procedia Eng.* **2014**, *94*, 18–24. https://doi.org/10.1016/j.proeng.2013.10.011.

(17) Bird, R. B.; Stewart, W. E.; Lightfoot, E. N. Chapter 14: Interphase Transport in Nonisothermal Systems. In *Transport Phenomena*; John Wiley & Sons, Inc., 2007; pp 423–425.

(18) Sze, S. M.; Ng, K. K. Chapter 1: Physics and Properties of Semiconductors-A Review. In *Physics of semiconductor devices*; Wiley-India: New Dehli, 2007; pp 7–68.
